# Silica-Based Nanoframeworks Involved Hepatocellular Carcinoma Theranostic

**DOI:** 10.3389/fbioe.2021.733792

**Published:** 2021-09-07

**Authors:** Yunxi Liu, Yue Chen, Weidong Fei, Caihong Zheng, Yongquan Zheng, Miao Tang, Ying Qian, Xiao Zhang, Mengdan Zhao, Meng Zhang, Fengmei Wang

**Affiliations:** ^1^ Department of Pharmacy, Women’s Hospital, Zhejiang University School of Medicine, Hangzhou, China; ^2^ School of Pharmacy, Zhejiang University of Technology, Hangzhou, China

**Keywords:** silica, nanoframeworks, hepatocellular carcinoma, diagnosis, therapy

## Abstract

Silica-based nanoframeworks have been extensively studied for diagnosing and treating hepatocellular carcinoma (HCC). Several reviews have summarized the advantages and disadvantages of these nanoframeworks and their use as drug-delivery carriers. Encouragingly, these nanoframeworks, especially those with metal elements or small molecular drugs doping into the skeleton structure or modifying onto the surface of nanoparticles, could be multifunctional components participating in HCC diagnosis and treatment rather than functioning only as drug-delivery carriers. Therefore, in this work, we described the research progress of silica-based nanoframeworks involved in HCC diagnosis (plasma biomarker detection, magnetic resonance imaging, positron emission tomography, photoacoustic imaging, fluorescent imaging, ultrasonography, etc.) and treatment (chemotherapy, ferroptotic therapy, radiotherapy, phototherapy, sonodynamic therapy, immunotherapy, etc.) to clarify their roles in HCC theranostics. Further, the future expectations and challenges associated with silica-based nanoframeworks were highlighted. We believe that this review will provide a comprehensive understanding for researchers to design novel, functional silica-based nanoframeworks that can effectively overcome HCC.

## Introduction

Hepatocellular carcinoma (HCC), comprising 75–85% of primary liver cancer, is one of the most diagnosed cancer worldwide. Nearly 900 thousand new cases and 830 thousand deaths due to HCC are reported each year globally (Sung et al., 2021). The 5-year survival rate of HCC is about 18%, making it the third leading cause of cancer after lung and colorectal cancers (Jemal et al., 2017). China contributes to the majority of HCC death each year (Kong et al., 2021), which is related to the high incidence of chronic infection with hepatitis B or C, substantial alcohol intake, obesity, smoking, and hepatic cirrhosis (Zheng et al., 2018). Hepatectomy, liver transplantation, transcatheter arterial chemoembolization, image-guided transcatheter tumor therapy, radiotherapy, chemotherapy, and immunotherapy are the treatment options for patients with HCC (Dimitroulis et al., 2017). Among them, hepatectomy is the first-line treatment for early stage HCC with a low mortality rate (<3%) after surgery (Kong et al., 2019a; Villanueva, 2019). However, usually, most patients with HCC are diagnosed at an advanced stage with rapid progression and poor prognosis (Pinyol et al., 2019; Teufel et al., 2019; Renne et al., 2020). Further, the poor selectivity of conventional chemotherapy accompanied by severe side effects and multidrug resistance (MDR) (Rudalska et al., 2014) seriously hinder the HCC treatment outcome. To make matters worse, the 5-year recurrence rate is up to 70% (Pinyol et al., 2019). Hence, developing novel scientific and technological ways for the early diagnosis, precise treatment, or diagnosis and treatment integration of HCC is in urgent need.

The development of nanotechnology promotes cancer treatment. More than 50 nanodrugs have been introduced for clinical use since 1995 (Chi et al., 2020). Further, several nanodrugs (such as ThermoDox, ALN-VSP, etc.) against HCC are in different stages of the clinical trial, which bring dawn to the patients with HCC (Tabernero et al., 2013; Lyon et al., 2017). In addition to liposome-based nanoformulations, polymers (Deng et al., 2021), dendrimers (Kianamiri et al., 2020), carbon-based nanovehicles (Yu et al., 2020), metal-organic frameworks (Hu et al., 2020), and mesoporous silica nanomaterials (MSNs) (Liu et al., 2020) have been developed as the nano theranostics for HCC. Among the abovementioned nanomaterials, MSNs are promising candidates for drug delivery due to their customizable pore/particle size, large surface area, high drug-loading capacity, and sustained/controlled release feature (Poonia et al., 2018). MSNs have been mainly synthesized by the modified Stöber method, also known as the sol-gel method. The templates, silica source, acid or base, and water are mixed in certain orders to form the silica sol, which turns into silica gel during the aging process. Calcination or the acidic solvent extraction process is then performed to obtain the MSN powder (Mohamed Isa et al., 2021). The properties of MSNs can be adjusted by controlling the added compounds or reaction conditions. MSNs can load different types of cargoes (hydrophilic or hydrophobic compounds, small molecules, large molecules, etc.) due to their customizable pore sizes and porous surfaces (Barkat et al., 2021). The easy-to-modify functional properties confer MSNs with site targeting as well as stimuli-responsive release characteristics (Zhao et al., 2019). For instance, our group constructed arginine-glycine-aspartic acid (RGD)-modified MSNs and polyacrylic acid-capped MSNs for targeted delivery and tumor microenvironment (TME)-sensitive release of antitumor drugs in HCC sites (Xiao et al., 2016; Fei et al., 2017). Both nanodrugs showed superior antitumor effects compared to the free drugs. Furthermore, MSNs can overcome MDR by co-delivering antitumor agents, thus providing a new approach to cancer therapy (Xue et al., 2017; Wu et al., 2018). Up to now, several reviews have summarized and analyzed the nano drug-delivery systems (nDDS) developed based on MSNs, fully demonstrating the potentials of this material in oncology diagnosis and treatment (Tao et al., 2020; Gao et al., 2020; Mohamed Isa et al., 2021). However, most of these reviews summarized MSNs as multi-functional drug carriers (Paris and Vallet-Regí, 2020; Mohamed Isa et al., 2021) or stimuli-responsive delivery platforms (Li et al., 2019a; Barui and Cauda, 2020; Živojević et al., 2021) for the treatment of tumors and other diseases such as osteosarcoma (Quadros et al., 2021), breast cancer (Poonia et al., 2018), glioblastoma (Nam et al., 2018), prostate cancer (Farina et al., 2018), atherosclerosis (Sha et al., 2021) and rheumatoid arthritis (Madav et al., 2020). Few of them have noticed that the framework of silica-based nanoplatforms (SNFs) can also take a leading part in the diagnosis and treatment of diseases. Hence, in this study, we reviewed the research advances of silica-based frameworks involving HCC theranostics.

The -Si-O-Si- bonds formed by the continuous hydrolysis of silica sources in an aqueous environment are the framework of conventional MSNs. With the extensive research performed in the past decade, researchers have found that the reconstruction or modification of the MSN skeleton not only maintain their superior drug-delivery characteristics but also confer MSNs with therapeutic effects against HCC. For instance, metal ions will hydrolyze to form metal-O bonds and dope into the framework of MSNs to generate metal-doped MSNs (Fei et al., 2020a). The spatial structure of the metal-doped MSNs showed similarity to that of conventional MSNs. Therefore, they still have high drug-loading efficiency as well as sustained/controlled drug release feature. Interestingly, the metal ions in novel SNFs can contribute to the diagnosis and treatment of HCC. In 2019, the research group constructed the manganese (Mn)-doped mesoporous silica nanocarrier (MnMSNs) (Tang et al., 2019). The -O-Mn-O- of MnMSNs disintegrated in the reducing cytoplasmic environment of HCC cells to release Mn^2+^, which could be used for the magnetic resonance imaging (MRI) diagnosis of HCC. Importantly, this process consumed glutathione (GSH) in HCC cells and destroyed the redox balance, and hence, this consumption played a synergistic effect against HCC with the loaded drugs. Apart from metal ions, MSNs can dope with small molecular compounds, such as dopamine (Liu et al., 2019c), carbon compounds (Lv et al., 2015), and phosphorous compounds (Fan et al., 2018), and the doped MSNs have high photothermal conversion efficiency and play a therapeutic effect on HCC. The metal-silica composite nanomaterials produced by the directional growth on the surface of metal nanomaterials can enhance the sensitivity of HCC cells to radiotherapy (Wang et al., 2017d). The existing reviews mainly describe the advantages of MSNs as chemotherapeutic drug carriers, however, a single chemotherapy approach is often unsatisfactory for HCC treatment. SNF-assisted tumor diagnosis and treatment strategies are expected to become the next research hotspot. To fully understand the role of SNFs and promote their clinical transformation, in this review, we summarized SNF-based diagnosis [nanochips for plasma biomarkers, MRI, positron emission tomography (PET), fluorescent imaging, ultrasound imaging, etc.] (Table 1), and treatment (chemotherapy, ferroptotic therapy, radiotherapy, phototherapy, sonodynamic therapy, and immunotherapy) for HCC (Table 2).

**TABLE 1:** The classification of functional SNFs for HCC diagnosis.

Imaging type	Functional SNFs	Size (nm)	Application	Ref
AFP detection	Ag/SiO_2_ nanoparticles and silica-coated Fe_3_O_4_ nanoparticles	35	Detect AFP for HCC	Gong et al. (2007)
AFP detection	Silicon Nitride Sensor Chips	40	Detect AFP for HCC	Kurihara et al. (2012)
Glycoprotein biosignature	SiO_2_@Ab and MNP@ConA	30–40	Uncover glycosylation biomarker for HCC	Dela Rosa et al. (2017)
Protein biomarker detection	Mag Au@IBA@MIP and Mag Au@ABA@MIP	300–400	Detect insulin for diabetes and AFP for HCC	Wang et al. (2020b)
Colorimetric detection	MIP particles based inverse opal hydrogel	300	AFP label-free detection	Wang et al. (2020a)
Colorimetric bioassay	SiO_2_ NPs-amplified Scadge-Diag	500	Detect HBsAg and AFP in clinical serum samples	Zhao et al. (2018)
MR imaging	Mn^2+^-doped SiO_2_ nanoparticles	25	HCC-specific MRI contrast agent	Kim et al. (2013)
	Arginine-rich manganese silicate nanobubbles (AMSNs)	6.2 ± 1.0	T_1_-weighted MRI	Wang et al. (2018)
Positron emission tomography	^68^Ga-labeled PET-SERRS nanoparticles	90	HCC imaging	Wall et al. (2017)
Photoacoustic imaging	Polydopamine-doped virus-like mesoporous silica coated graphene oxide nanosheets	500	HepG_2_-bearing tumor diagnosis	Liao et al. (2020)
	Hyaluronic Acid-Conjugated Silica Nanoparticles	2	Diagnosis of liver diseases	Lee et al. (2018)
Fluorescence detection	Carboxyl-modified fluorescein isothiocyanate (FITC)-doped silica nanoparticles	75.47 ± 2.52	Detection of HepG_2_ hepatoma cells	Hu et al. (2017)
	PEG modified RuBpy-doped silica fluorescent nanoparticles	60	Recognition and imaging of human HCC cells	Chen et al. (2014)
Ultrasound imaging	GPC-SiNP	350	Ultrasound molecular imaging for human HCC cells	Di Paola et al. (2017)

**TABLE 2:** The classification of functional SNFs for hepatocellular carcinoma therapy.

Therapy strategies	Functional SNFs	Size (nm)	Cargoes	Mechanism-of-action	Ref
Chemotherapy	Berberine-capped silica nanoparticles	54.4 ± 2.8	Ber	Conjugated berberine on the nanosurface (dendritic effect) led to the enhanced apoptotic activity	Halimani et al. (2009)
	YMSNs-NBC-Cyt c-NBC-LA	163.3 ± 26.84	DOX	The released Cyt c could induce the mitochondrial apoptosis pathway and initiated DOX release to induce combined chemotherapy	Pei et al. (2018)
	Titanocene derivatives immobilized nanostructured silica materials	Length: 850; Width: 400	None	Titanocene derivatives acted as the entire nanoparticulated system to induce chemotherapy	Gómez-Ruiz et al. (2018)
	ZnPc/DOX@MSN	176	DOX	ZnPc acted as photosensitiser to induce PDT and DOX induced cytotoxicity	Wong et al. (2020)
	FA-JNPs@ICG	280–380	None	ICG was employed as the effector for photothermal therapy and the subsequently released silver ions induced cytotoxicity	Wang et al. (2017c)
Ferroptosis induction	Arginine-rich manganese silicate nanobubbles	14.6	DOX	-Mn-O- bond cleavage consumed GSH and induced ferroptosis	Wang et al. (2018)
	Folate-PEG manganese doped MSNs	120	DHA	-Mn-O- bond cleavage consumed GSH and generated ROS to induce ferroptosis	Fei et al. (2020a)
Radiotherapy	Au-mesoporous silica Janus nanoparticles	Length:200–250; width: 100–120	DOX	Au NPs emerged as radiosensitizer to induce radiotherapy and DOX induced cytotoxicity	Wang et al. (2017d)
	TPZ-loaded Janus gold triangle-MSNs	Gold head: 60; silica linker: 200	TPZ	Au NPs emerged as radiosensitizer to induce radiotherapy as well as PTT and TPZ induced cytotoxicity	Wang et al. (2019)
	Ber-loaded Janus gold MSNs	Gold nanorod: 50–60/10–15; Silica stick: 200–250/100–120	Ber	Au NPs emerged as radiosensitizer to induce radiotherapy as well as PTT and Ber induced cytotoxicity	Li et al. (2019b)
Phototherapy	GdOF:Ln@SiO_2_-ZnPc-CDs-FA UCL microcapsules (UCMCs)	293	DOX	The codoped Yb/Er/Mn in GdOF trandfered energy to ZnPc to induce PDT while carbon dots generated thermal effect, and DOX induced chemotherapy	Lv et al. (2015)
	ZnPc-DOX conjugated MSNs	100	DOX	ZnPc generated ^1^O_2_ to induce photodynamic conversion and DOX induced chemotherapy	Wong et al. (2017)
	TLS11a-decorated BPQDs-hybridized mesoporous silica framework	52.3 ± 5.7	Pt nanoparticles	Black phosphorus doped MSNs generated ^1^O_2_ to induce photodynamic conversion	Lan et al. (2019)
	pGSNs-DOC	120	DOC	The gold nanoshell induced photothermal effect and DOC induced chemotherapy	Liu et al. (2011)
	ConA-decorated silica–carbon hollow spheres	300	DOX	Silica-carbon hollow spheres induced photothermal and DOX induced cytotoxicity	Chen et al. (2018)
	Core-shell structured graphene oxide/mesoporous silica@alginate	Not found	MTX	Graphene oxide induced photothermal conversion and DOX induced cytotoxicity	Li et al. (2021a)
	Light-activated core-shell structured Au nanorod/mesoporous silica nanocontainer	224.2	DOX	Au nanorod induced photothermal conversion and DOX induced cytotoxicity	Yang et al. (2021)
Sonodynamic therapy	DOX@HMONs-PpIX-RGD	40	DOX	HMONs with molecularly organic-inorganic hybrid framework generated ^1^O_2_ to apoptosis of HCC cells	Li et al. (2018)
Immunotherapy	MSN-SP-LPS	167	DOX&LPS	LPS activated immune response and DOX induced cytotoxicity	Dong et al. (2017)

## SNF-Based HCC Diagnosis

Better prognosis is usually observed in patients with HCC who are in their early stages when the liver function is still preserved and the patients are asymptomatic. Hence, the early diagnosis of HCC can significantly increase the survival rate and improve the prognosis of these patients (Etzioni et al., 2003; Ayuso et al., 2018). The diagnosis of HCC mainly consists of plasma biomarker detection and imaging examinations like MRI, PET, and ultrasound. However, the disadvantages of these diagnostic modalities namely low sensitivity, high false-positive rates, non-specific features hinder their clinical use (Pinero et al., 2020). Thus, novel diagnostic agents and instruments for HCC are urgently needed. Silica-based nanomaterials are of particular interest for HCC diagnosis due to their unique structural and functional properties (Mochizuki et al., 2021). Several studies have attempted to find more precise ways for the early diagnosis of HCC by integrating silica-based nanoframeworks with existing diagnostic technology (Table 1).

### SNF-Based Nanochips for the Identification of HCC Plasma Biomarkers

The recognition of HCC biomarkers in the plasma is vital for its early clinical diagnosis. Alpha-fetoprotein (AFP) is the most commonly detected tumor-associated protein and is used for the early diagnosis of HCC (Sauzay et al., 2016). However, the current diagnostic approaches have limited detection sensitivity and are time-consuming or require complex instruments, making the development of highly sensitive detection technologies an urgent requirement for detecting serum biomarkers present in very low concentrations (Ayuso et al., 2018; Singh et al., 2020). A sandwich immunoassay based on surface-enhanced resonance Raman (SERR) spectroscopy was developed that exhibited high sensitivity and specificity to human AFP (Gong et al., 2007). This system was composed of Ag/SiO_2_ nanoparticles-loading rhodamine B isothiocyanate dye as the SERR-scatting (SERRS) tags and silica-coated magnetic nanoparticles as the immobilization matrix and separation tool. Polyclonal antibody and monoclonal antibody were chemically modified onto the surfaces of Ag/SiO_2_ nanoparticles and silica-coated magnetic nanoparticles respectively, using glutaraldehyde as a crosslinker. The monoclonal antibody-modified silica-coated magnetic nanoparticles were first mixed with PBS buffer containing the AFP antigen and 1% bovine serum albumin and subsequently incubated with polyclonal antibody-modified Ag/SiO_2_ nanoparticles. The sandwich immunocomplexes were separated from the solution under a constant magnetic field and subjected to Raman analysis. An augmented Raman signal was observed as a result of the fluorescence quenching and the Raman-enhancement ability of silver. Moreover, the detection results of the AFP antigen at diverse concentrations showed that the sandwich immunoassay had high sensitivity and specificity for AFP recognition with a detection range of 11.5 pg/ml to 0.12 μg/ml (Gong et al., 2007). Inspired by the arginine selective conductometric biosensor based on arginase and urease (Soldatkina et al., 2018), a conductometric immunoassay based on the bienzyme (arginase and urease)-functionalized nanometer-sized silica beads was developed to detect AFP (Liang et al., 2018). First, the ureases were doped into silica nanoparticles *via* the reverse micelle method. Then, the arginase-labeled anti-AFP secondary antibodies were coupled to the urease-doped nanosilica particles. The anti-AFP capture antibodies were coated on the microplates and the sandwich-type immunoreaction was carried out by adding the AFP-containing sample and arginase-coated nanometer-sized urease-doped silica beads. Finally, the introduced L-arginine was enzymolyzed to L-ornithine and urea by the arginase, and the produced urea was subsequently decayed into ammonia and bicarbonate under the urease enzymatic reaction, resulting in the changed conductivity of the solution that could be quantitatively monitored. The nanometer-sized silica beads increased the loading amounts of enzymes based on their high surface-to-volume ratio and quantum-size effect and further amplify the detection signal. The immunosensing system showed a highly sensitive conductometric response to AFP with a linear detection range of 0.01–100 ng/ml and a detection limit of 4.8 pg/ml. Importantly, the reproducibility and precision with a relative standard deviation of <15% and specificity with no response to other cancer biomarkers such as CA125, PSA, and hCG, etc., were satisfactory for AFP detection in HCC patients (Liang et al., 2018).

**FIGURE 1:**
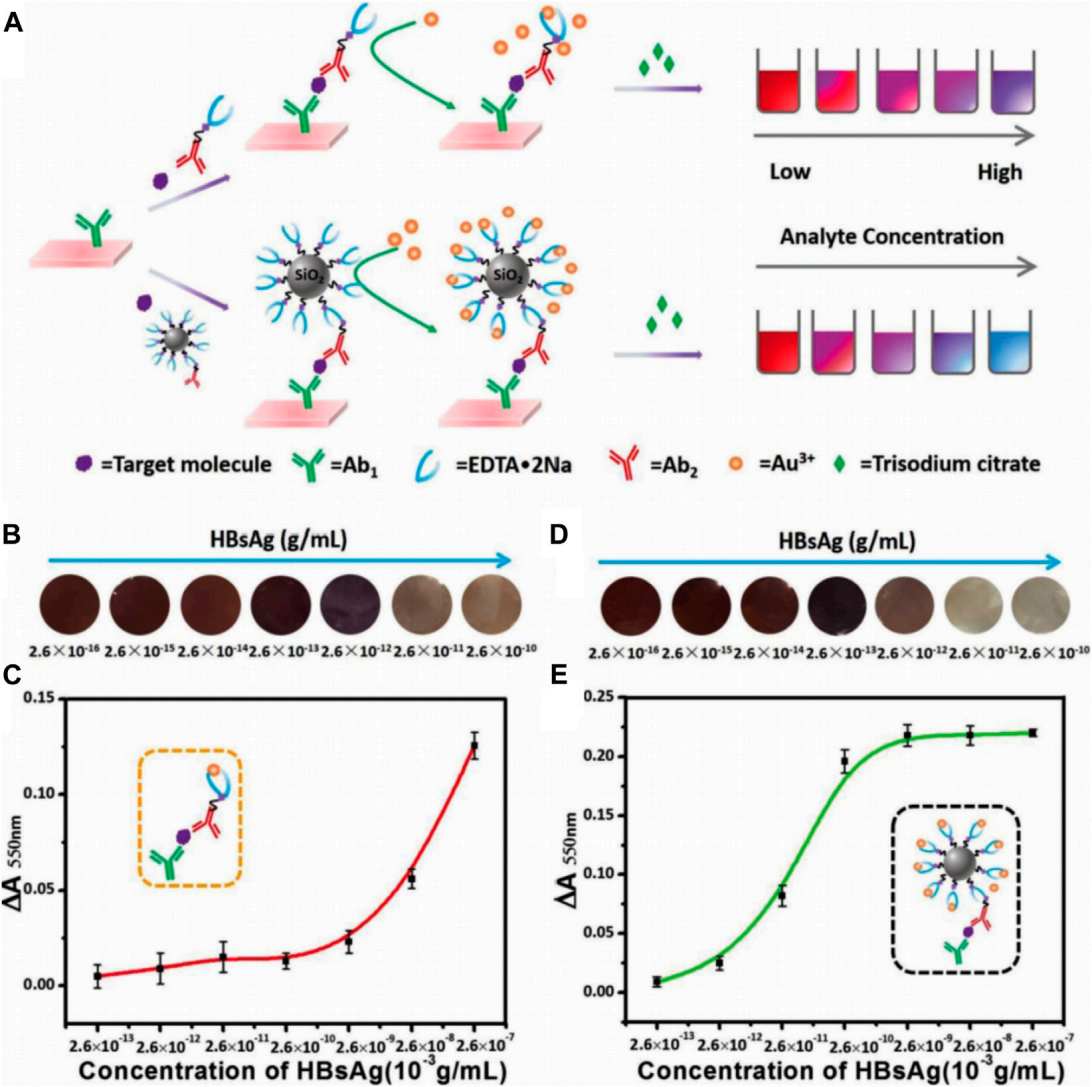
**(A)** Schematic illustration of Scadge-Diag and SiO_2_ NPs-amplified Scadge-Diag. **(B,D)** Practical detection of HBsAg by naked-eye visualization and **(C,E)** plotting absorbance values of AuNPs collected at 550 nm versus varying concentrations of HBsAg based on **(B,C)** Scadge-Diag and **(D,E)** SiO_2_ NPs-amplified Scadge-Diag, respectively. Reprinted with permission from Zhao et al. (2018). Copyright (2018) John Wiley and Sons.

To overcome the deficiency of enzyme-based immunoassays that depend on the enzyme catalytic activity, which are determined by thermophilic scope and stability, an enzyme-free metal sequestration of a complexing agent to dominate the gold nanoparticle generation (called Scadge) strategy was carried out (Zhao et al., 2018). The detection signals originated from the *in situ* generated gold nanoparticles (AuNPs) that were regulated by the metal chelator ethylenediaminetetraacetic acid disodium salt (EDTA·2Na). This proposed diagnostic mode was dubbed Scadge-Diag, and silica nanoparticles (SiO_2_ NPs) were utilized to further amplify the detection sensitivity *via* chemically grafting EDTA·2Na onto the NP surfaces (Figure 1). The Scadge-Diag system exhibited ultrasensitivity toward HBsAg and AFP in the presence of auxiliary SiO_2_ NPs, with a detection limit of 2.6 × 10^–15^ g/ml for HBsAg and 2.5 × 10^–19^ g/ml for AFP.

Novel diagnostic instruments are important for effectively detecting biomarkers. A microfluidic reflectometric interference spectroscopy (RIfS) system based on a halogen light source and carboxylate groups-modified silicon nitride sensor chips was developed for the label-free AFP detection (Kurihara et al., 2012). The anti-AFP antibody was modified onto the surface of a silicon nitride chip that was immobilized on the Si wafer. This RIfS-based sensor had high reproductivity with a coefficient variation of 5.7% and no cross-reaction with other proteins such as human serum albumin, thus exhibiting the potential as an effective biomarker detection tool. The development of silica-based diagnostic approaches and instruments for the early detection of plasma biomarkers can facilitate the prevention and early diagnosis of HCC, and further improve HCC management.

### SNF-Based MRI of HCC

**FIGURE 2:**
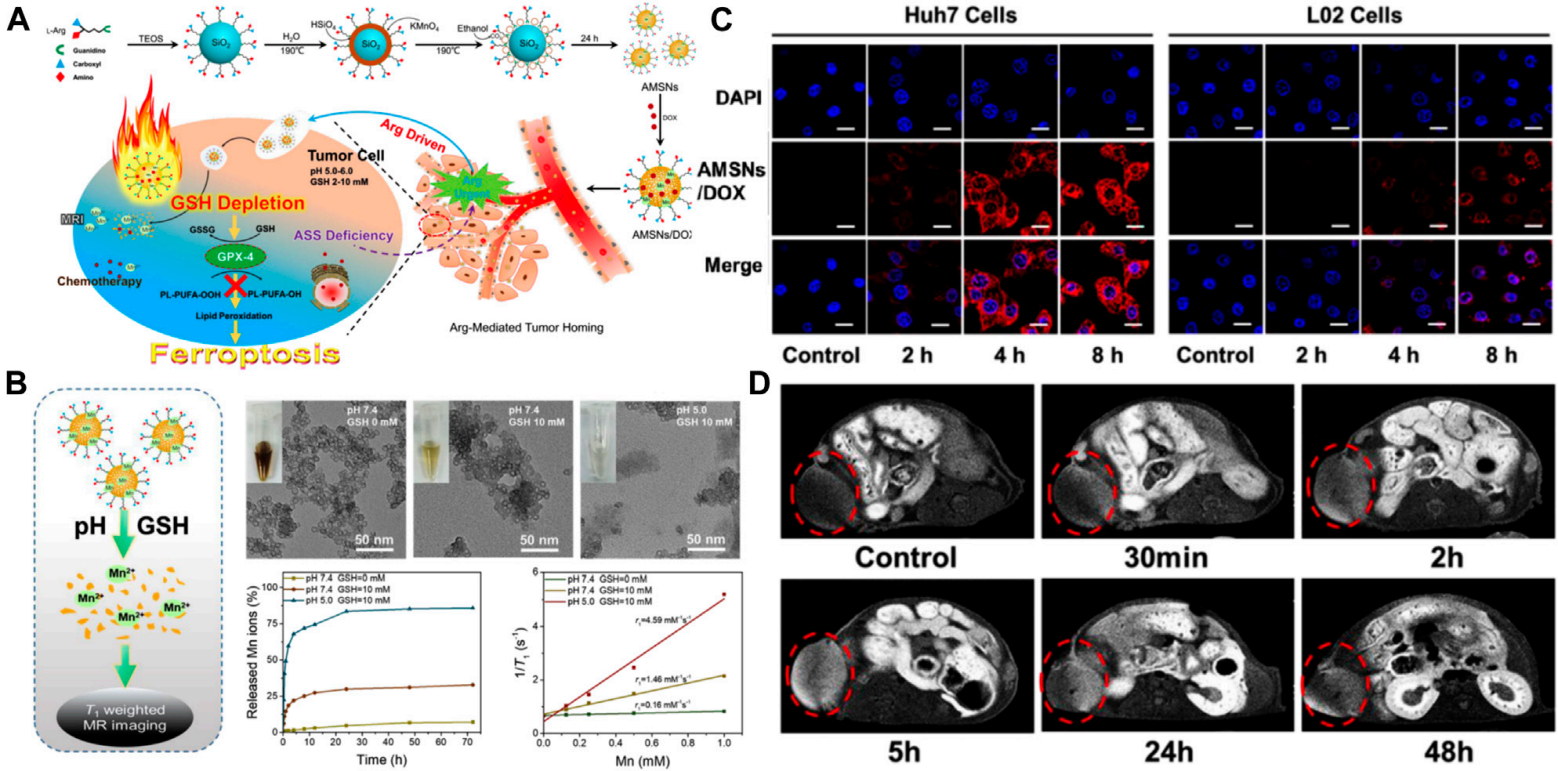
**(A)** Schematic illustration of the designed synthesis of the arginine-rich manganese silicate nanobubbles (AMSNs) as well as the *in vivo* tumor homing after blood circulation. **(B)** Schematic illustration of the biodegradation of AMSNs in low pH and high GSH microenvironment and accumulated release profiles of Mn ions in various pH (7.4, 5.0) and GSH concentrations (0 and 10 mM). **(C)** Confocal laser scanning microscope (CLSM) images of the Huh7 liver cancer cells and L02 normal liver cells after incubation with AMSNs/DOX for various periods. **(D)** *In vivo* T1-weighted MRI of tumor-bearing mice after the intravenous administration of AMSNs for different periods. Reprinted with permission from Wang et al. (2018). Copyright (2018) American Chemical Society.

MRI exhibiting a high spatiotemporal resolution and excellent soft-tissue contrast is frequently used as a noninvasive imaging modality in clinical diagnosis. Gadolinium chelate has been regularly used in T1-weighted MRI but its use has become limited because of nephrotoxicity, nonspecificity, and poor quality of images (Canavese et al., 2008; Kim et al., 2018). To develop a more sensitive, reliable, and safer MRI contrast agent for use in HCC-specific MRI, Kim et al. synthesized Mn^2+^-doped SiO_2_ nanoparticles (Mn-SiO_2_) (Kim et al., 2013). X-ray diffraction results of the nanospheres showed that Mn^2+^ ions were homogenously scattered in the silica structure. The inert silica structure not only improved nanoparticle safety by hindering the release of Mn^2+^ ions in the bloodstream but also detected HCC in MRI by taking advantage of the brightly enhanced MRI due to the release of Mn^2+^ under acidic conditions. The signal MRI intensities depended on Mn^2+^ concentration. A gradual but significant signal enhancement was observed in the T1-weighted signal intensity of normal tissues, which peaked at 6 h after injection. On the other hand, a different enhancement behavior was observed when HCC tissues were used and this behavior was constant for the first 6 h and enhanced brightly with the uptake of Mn^2+^ after 6 h, thus achieving a higher liver-to-HCC contrast ratio. This enhancement phase distinction between HCC and normal tissues helped in producing highcontrast enhancement HCC pictures and achieved reliable diagnosis of liver lesions over normal tissue, thus demonstrating the unique MR contrast-enhancing characteristics of Mn-SiO_2_. In another study, Wang et al. synthesized arginine-rich manganese silicate nanobubbles (AMSNs) for GSH depletion-induced ferroptosis and tumor-specific MRI (Figure 2A) (Wang et al., 2018). As shown in Figure 2B, the–Mn–O– bonds in AMSNs can be cleaved and the release of free Mn^2+^ ions can be triggered by a decrease in pH or the abundance of GSH in the tumor intracellular microenvironment. Transmission electron microscope (TEM) images showed the gradual structural degradation of AMSNs as pH decreased and the GSH concentration increased. In addition, the accumulated release amount of Mn^2+^ increased along with the degradation of AMSNs under acidic and high GSH conditions. In this study, arginine (Arg) was considered a targeting moiety for HCC theranostics. HCC cells cannot produce Arg themselves due to the lack of argininosuccinate synthetase (Ensor et al., 2002). After Huh7 liver cancer cells and normal liver cells were incubated with AMSNs and DOX (Figure 2C), stronger fluorescence intensity was observed in the Huh7 liver cancer cells than in normal liver cells, indicating the selective uptake of AMSNs by HCC cells. In the tumor-bearing mice (Figure 2D), the signal intensity of T1-weighted MRI at the tumor site enhanced at 2 h after injection and reached the peak until 5 h after injection, owing to the accumulation of the released Mn^2+^ in HCC tissues. This enhancement-phase distinction between cancer cells and surrounding tissue help in producing high-contrast enhancement HCC pictures and led to the accurate diagnosis of liver lesions over normal tissues. More studies have also confirmed that Mn^2+^ doped MSN can be used as a highly sensitive MRI diagnostic agent (Chen et al., 2012; Chen et al., 2015b; Fei et al., 2020a). In general, SNFs may be effective and less toxic hepatoma-specific MRI contrast agents.

### SNF-Based PET of HCC

**FIGURE 3:**
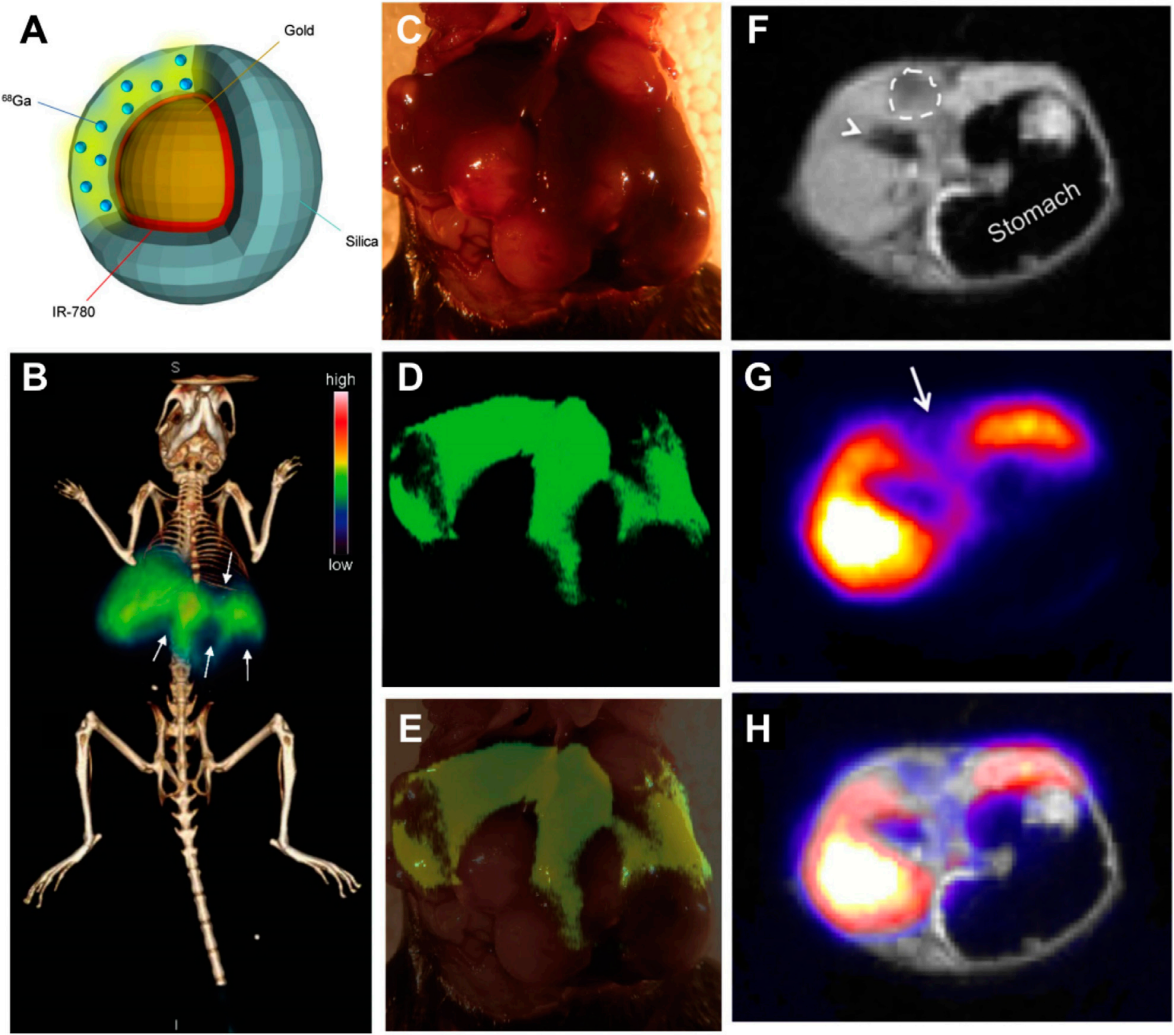
**(A)** Schematic illustration of the PET-SERRS nanoparticle. **(B)** PET-computed tomography image of a tumor-bearing mouse after injection with PET-SERRS nanoparticles. **(C)** The intraoperative white light image of the liver from the mouse imaged in **(A)**. **(D)** SERRS imaging of the liver (high SERRS signal) and location and extent of the tumors (signal voids) and **(E)** overlay of photograph and SERRS map. **(F–H)** PET-MRI of HCC using PET-SERRS nanoparticles. Reproduced with permission from Wall et al. (2017). Copyright (2017) Ivyspring International Publisher.

PET is a noninvasive nuclear imaging technique that can identify metabolic processes in the human body with high sensitivity. The underlying principle of PET is the detection of gamma rays emitted indirectly by radionuclides depending on the different metabolic states of tumors and normal tissues (Lu et al., 2019). Currently, chelator-based radionuclides are still widely used for synthesizing radio-labeled nanoparticles. However, considering the issues about the accessibility of specific chelation agents, undesired changes in pharmacokinetics, and long-term integrity after chelation, chelator-free labeling nanoparticles were synthesized (Chen et al., 2015a; Wall et al., 2017). Among them, Wall et al. fabricated chelator-free radiolabeling of SERR scatting (SERRS) nanoparticles called ^68^Ga-labeled PET-SERRS nanoparticles (Wall et al., 2017). This nanoparticle consisted of a 60 nm diameter gold core, a Raman reporter dye layer, and a silica shell distributed with ^68^Ga of 30 nm (Figure 3A). The silica shell was necessary for chelator-free radiolabeling and sulfur was added to silica surfaces to stabilize the radiolabeling of radiometal ions. Radionuclides were embedded throughout the silica shell and the SERRS nanoparticles were obtained that exhibited distinct spectral signatures and were stable regardless of photobleaching. Since the nanoparticles accumulated in the reticuloendothelial system (RES), they could be used particularly for HCC imaging. In tumor-bearing mice, the liver PET-CT image revealed clear filling defects (Figure 3B). These defects matched the tumor sizes and locations after surgically exposed (Figure 3C), intraoperative SERRS imaging (Figures 3D,E), or MRI scanning (Figures 3F–H), successfully delineating the presence of HCC. The PET-SERRS nanoparticles combined the whole-body imaging of PET and the high resolution of SERRS imaging, enabling superior visualization sensitivity of as little as 100 μm, and the possibility of a rapid preoperative roadmap and precise intraoperative surgical guidance. Thus, silica-based nanoplatforms were successful in photobleaching radionuclides in PET imaging and broadened their clinical use in HCC diagnosis.

### SNF-Based Photoacoustic Imaging of HCC

**FIGURE 4:**
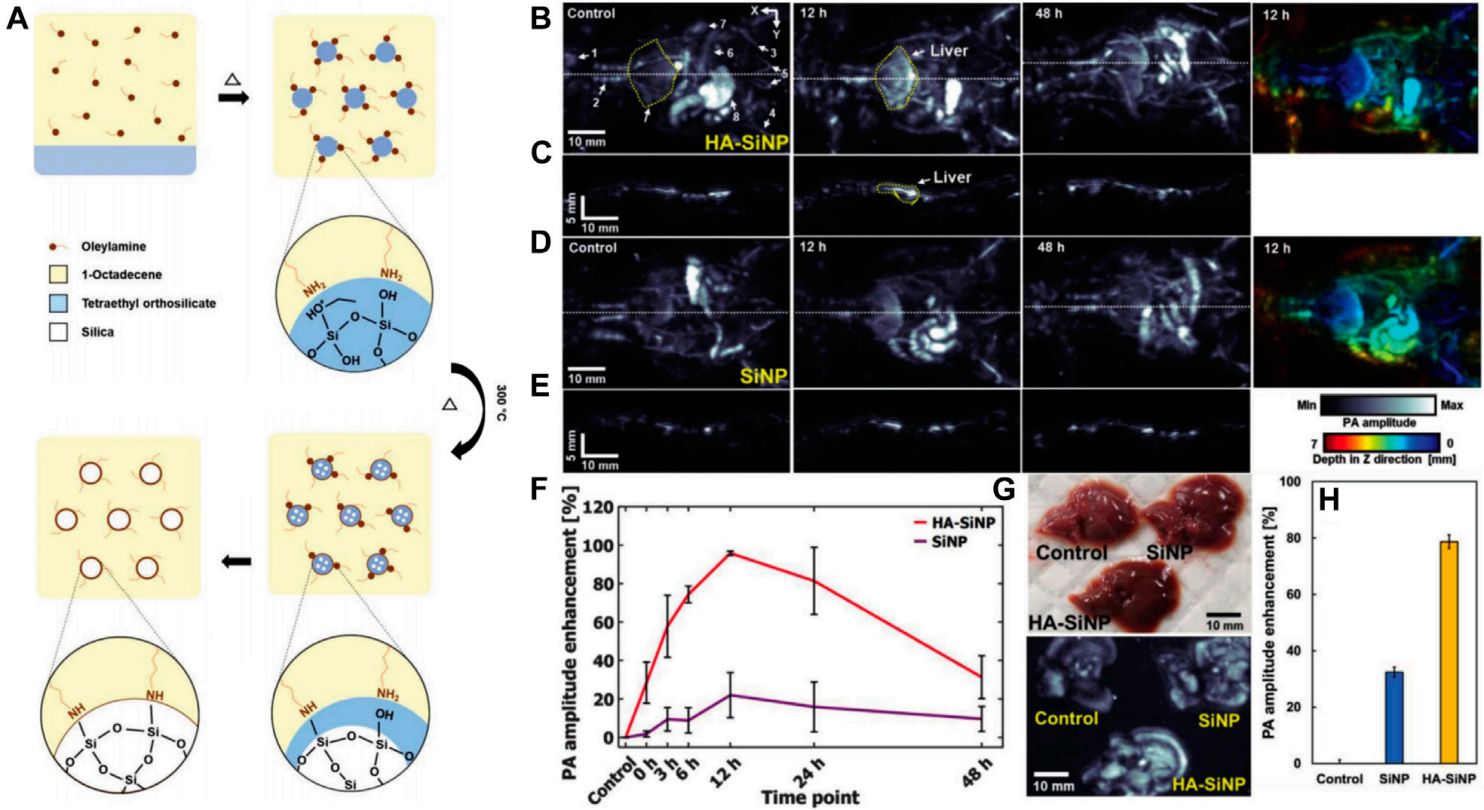
**(A)** Preparation of SiNP. **(B)** *In vivo* PA MAP images and a depth-color encoded PA image of mouse whole body after HA–SiNP conjugate injection. **(C)** PA cross-sectional images at the white dashed line in **(B)**. **(D)** *In vivo* PA MAP images and a depth-color encoded PA image after SiNP injection. **(E)** PA cross-sectional images at the white dashed line in **(D)**. **(F)** PA amplitude profile in the liver region from before injection to after HA–SiNP conjugate and SiNP injection (*n* = 3). **(G)** Photograph and *ex vivo* liver PA MAP image 12 h after HA–SiNP conjugate and SiNP injection. **(H)** *Ex vivo* PA amplitude enhancement at HA–SiNP conjugate and SiNP injected liver (*n* = 3). Reprinted with permission from Lee et al. (2018). Copyright (2018) John Wiley and Sons.

Photoacoustic imaging (PAI) integrating optical imaging with a high contrast rate and ultrasonic imaging with a high penetration depth has garnered considerable attention in the field of medical science. At the core of this technique is a near-infrared (NIR) laser. When the energy is absorbed and converted into heat, a wind-band ultrasonic emission is produced. The resulting ultrasonic waves can be analyzed and converted into an image (Johnson et al., 2019; Liu et al., 2019a). Liao et al. constructed polydopamine-doped virus-like mesoporous nanoparticles (PVMSNs) coated with reduced graphene oxide (rGO@PVMSNs) nanocomposites (Liao et al., 2020). These nanocomposites not only had the inherent properties of the mesoporous material but also a virus-like structure. The virus-like mesoporous nanoparticles gained the extra capability of invading cells with prolonged blood circulation times than traditional MSNs (Wang et al., 2017b). Moreover, the rGO@PVMSNs exhibited excellent photothermal properties *in vitro*. Due to the enhanced cellular uptake by HepG_2_ cells, the photoacoustic (PA) image in the tumor region was more obvious after rGO@PVMSN injection for 24 h, suggesting that rGO@PVMSNs could be a potential photoacoustic imaging contrast agent for biomedical imaging. In another study, novel hyaluronate-silica nanoparticle (HA-SiNP) conjugates were synthesized (Figure 4A) (Lee et al., 2018). The acoustic properties of SiNP, and the high biocompatibility and biodegradability of HA conferred the synthesized HA-SiNP conjugates with high liver specific-targeting efficiency, strong optical absorbance near-infrared windows, and excellent biocompatibility and biodegradability. After endocytosis specifically into HCC cells, the HA-SiNP conjugates presented an enhanced PA amplitude than that in the control group and a PA amplitude 4.4 times greater than that of SiNP (Figures 4B–H). These two SNFs made PAI useful in HCC diagnosis with high-resolution anatomical and functional information in deep tissues like the liver.

### SNF-Based Fluorescence Imaging of HCC

**FIGURE 5:**
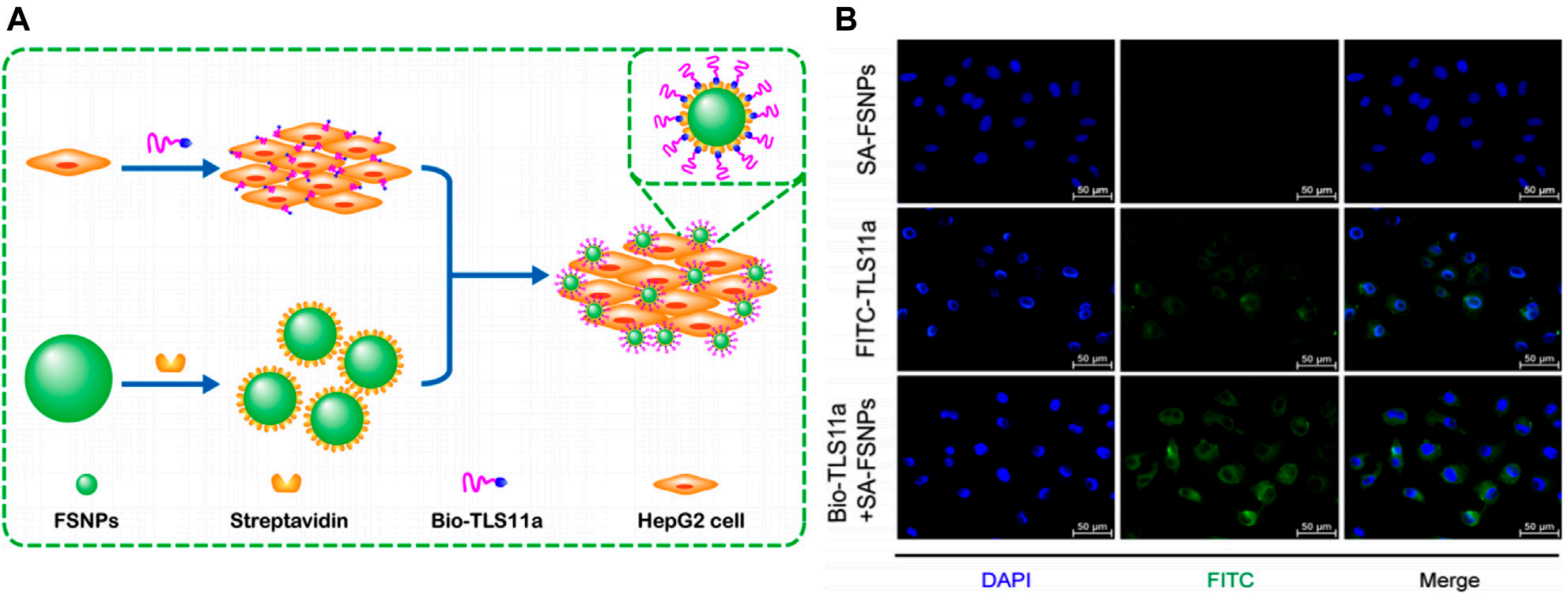
**(A)** Schematic illustration of highly sensitive detection of HepG_2_ hepatoma cells using a biotin-conjugated aptamer (Bio-TLS11a) and streptavidin-conjugated fluorescent silica nanoparticles (FSNPs). **(B)** Fluorescence micrographs of HepG_2_ cells after incubation with SA-FSNPs or FITC-TLS11a or the combination of both. Reprinted with permission from Hu et al. (2017). Copyright (2017) Springer.

Fluorescence imaging using fluorescent dyes as markers has been widely used in cytological and histological analyses. However, the disadvantages of fluorescent dyes including fluorescence bleaching and poor biocompatibility have limited their further development and clinical applications (Tan et al., 2016). To overcome these limitations, biofunctional molecules such as antibodies or aptamers were conjugated with fluorescent nanoparticles (Smith et al., 2007). Particularly, dye-doped silica nanoparticles were outstanding because they exhibited good biocompatibility and resistance to photobleaching, and their surface could be easily modified (Wang et al., 2013). Hu et al. developed carboxyl-modified fluorescein isothiocyanate (FITC)-doped silica nanoparticles (SA-FSNPs) conjugated with streptavidin (SA) (Hu et al., 2017). The functionalized silica nanostructure not only inherited the fluorescence properties of the FITC dye but was also able to emit stronger and photobleach-resistant fluorescent signals. The underlying principle of this nanosystem was based on the biotin-labeled aptamer TLS11a (Bio-TLS11a) and streptavidin-modified FSNPs. Bio-TLS11a were labeled with hepatoma cells and physically separated from FSNPs (Figure 5A). After incubation with HCC cells, the bio-TLS11a and SA-FSNP combination emitted a stronger fluorescence signal than SA-FSNPs or bio-TLS11a alone (Figure 5B). Moreover, this fluorescence remained visible for 10 min, whereas the fluorescence of FITC-TLS11a lasted only for 2 min. SA-FSNPs detected aptamer-labeled HCC with good sensitivity and excellent specificity and emitted strong, photobleach-resistant fluorescent signals without the obvious noxious effect in cells or *in vivo*. In another study, Yan et al. developed PEG-modified RuBpy-doped silica nanoparticles that coupled with avidin (Chen et al., 2014). The dye-doped silica nanoparticles had excellent photostability. After incubation with HepG_2_ cells, these nanoparticles effectively recognized the tumor marker carcinoembryonic antigen with a magnified fluorescence signal. The SNFs exhibited enhanced sensitivity, recognition efficiency, and photostability of fluorescence imaging, and became ideal for HCC diagnosis.

### SNF-Based Ultrasound Imaging of HCC

Ultrasound (US) is an indispensable imaging tool of clinical diagnosis. Developing novel ultrasound contrasts is important to enhance the accuracy of clinical ultrasound diagnosis (Hunt and Romero, 2017). Nanoparticles have been widely studied as targeted imaging and therapeutic agents because of their unique size and high tissue extravasation. Solid nanoparticles such as silica and polystyrene particles were studied for their potential of enhancing the ultrasound effect (Liu et al., 2006). The *in vitro* results of agarose gel imaging showed that silica and polystyrene particles increased the imaging signal in a concentration-dependent manner, and the particle size had a considerable effect on the image brightness. After intravenous injection, the silica nanoparticle-mediated ultrasound signal in the liver parenchyma of mice increased over time due to the aggregation of silica nanoparticles in the lysosomes of Kupffer cells (Liu et al., 2006). Based on the potential for enhancing ultrasonography, glypican-3 protein (GPC-3) ligand peptide-modified silica nanoparticles loaded with FITC were designed for the targeted ultrasound molecular imaging of HCC cells mediated by GPC-3 overexpression on the cell surface (Di Paola et al., 2017). The *in vitro* results showed that the nanoparticles significantly enhanced the ultrasound signal; and at the ultrasound detection concentration they could effectively bind to the HepG_2_ cells and be further uptaken. MSNs with a high specific surface area and hydrophobic surfaces could be nanobubble precursors and *in situ* generated microbubbles after appropriate stimuli to overcome the short lifetime of microbubbles that are used as ultrasound contrast agents (Jin et al., 2017). Perfluorodecyltriethoxysilane-modified MSNs (F-MSNs, porous and superhydrophobic) generated more durable microbubbles lasting for at least 30 min at a mechanical index of 1.0 (FDA safety guidance, MI ≤ 1.9), whereas lipid microbubbles lasted for just about 5 min. F-MSNs exhibited more pronounced and stronger ultrasound contrast signals than other nanoparticles such as MSNs (porous and hydrophilic), trimethylchlorosilane-modified MSNs (M-MSNs, porous and low hydrophobic), and perfluorodecyltriethoxysilane-modified silica nanoparticles (P-SS, superhydrophobic but nonporous) (Jin et al., 2017). The strategy of *in-situ* producing microbubbles from the interfacial nanobubbles (INBs) adsorbed onto the superhydrophobic surfaces of MSNs can provide potentials for developing the novel silica-based ultrasound contrast agents for HCC diagnosis. These SNFs were superior with enhanced ultrasound signals and a longer lifetime.

## SNF-Based HCC Therapy

### SNF-Based Chemotherapy

Cancer pharmacological treatment has revolved rapidly since their invention (Falzone et al., 2018), and chemotherapy and targeted therapy have been the major approaches for cancer treatment (Zhong et al., 2021). However, their clinical use was limited because of severe systemic adverse reactions and multidrug resistance. Generally, MSNs were used as delivery carriers of chemotherapeutic agents such as sorafenib (Zhao et al., 2017), doxorubicin (DOX) (Ye et al., 2018), cisplatin (Wang et al., 2017e), and curcumin (Kong et al., 2019b) to improve their therapeutic effects and reduce the side effects by enhancing the bioavailability of hydrophobic drugs and tumor-cell targeting. However, some disadvantages were also observed such as the sudden release or their release in circulation. Conjugating drugs into the silica skeleton could address these problems and confer other advantages such as increasing drug loading, drug stability, specific site targeting, and sustained release profile (Halimani et al., 2009; Pei et al., 2018; Wong et al., 2020).

Pei et al. immobilized cytochrome c (Cyt c) onto the surface of yolk-shell mesoporous silica nanoparticles (YMSNs) *via* boronic ester bonds to fabricate the tumor-targeted H_2_O_2_-responsive Cyt c/DOX co-delivery system (YMSN-NBC-Cyt-c-NBC-LA@DOX) (Pei et al., 2018). YMSNs, as a novel form of MSNs, were capable of a relatively high drug loading for chemotherapeutic agents. Moreover, these modifications conferred these novel MSNs with tumor-targeting features, and these MSNs could temporarily shield the bioactivity of Cyt c and further prevent DOX from premature release. In the presence of high levels of reactive oxygen species (ROS) in the TME, the boronic ester could be rapidly cleaved and the Cyt c would be removed from the surface. This process could restore the bioactivity of Cyt c and readily initiate the subsequent DOX release. The *in vitro* study revealed the cytotoxicity effect of H_2_O_2_-responsive Cyt c delivery system (YMSN-NBC-Cyt-c-NBC-LA) toward HepG_2_ cells in a dose-dependent manner. Regarding the *in vivo* effect, the tumors of mice treated with YMSN-NBC-Cyt-c-NBC-LA@DOX were inhibited, whereas the tumors of the saline-treated group grew rapidly. In another study, Halimani et al. synthesized a series of novel berberine-capped silica nanoparticles bearing covalently linker chain lengths from C_2_ to C_6_ (Halimani et al., 2009). With the existence of CuSO_4_ and sodium ascorbate, these nanoparticles were fabricated by the click reaction between alkyne-bearing silica nanoparticles and the azidoberberine analogs. A higher surface density of berberine in the silica-berberine combination was achieved with a hexyl linker than an ethyl linker. Multivalent or dendritic effects were observed when therapeutic molecules on the nanoparticles reacted with the interacting partners inside the cell, thereby enhancing the therapeutic effect. In accordance with these effects, the *in vitro* study showed that all nanoconjugates exhibited higher inhibition with the IC_50_ values of 0.2–0.27 μm than free berberine (IC_50_ = 1.4 μm) toward HepG_2_ cells 24 h after treatment. Moreover, there was an increase in the cytotoxic effect against HCC of these nanoparticles with an increase in the linker chain length. The mechanism underlying the phenomena was the cell cycle arrest and the selective apoptotic cell death, suggesting the potential role of the spacer chain in strengthening the pharmacological effects of these nanoparticles.

**FIGURE 6:**
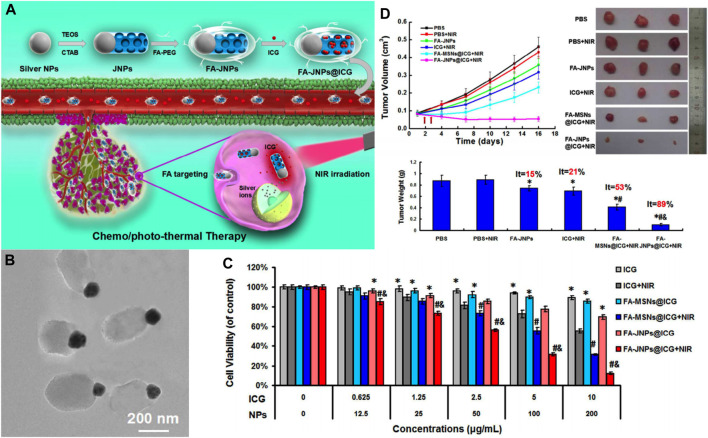
**(A)** Schematic illustration of the FA-targeted Janus nanoplatform with the NIR irradiation-triggered release behavior of ICG and silver ions for synergistic liver cancer chemo/photothermal therapy. **(B)** TEM images of the FA-JNPs. **(C)** Cells viability of SMMC-7721 cells incubated with various concentrations of ICG, FA-MSNs@ICG, or FA-JNPs@ICG with or without NIR irradiation for 24 h (808 nm, 1 W cm^−2^, 5 min). **(D)** *In vivo* tumor inhibition evaluation of the chemo/photothermal therapy. Reprinted with permission from Wang et al. (2017c). Copyright (2017) American Chemical Society.

In addition to covalently grafting small-molecule drugs into the framework of MSNs, metal-silica nanoparticles exhibited excellent chemotherapeutic effects. Wang et al. engineered an FA-targeted, indocyanine green (ICG)-loaded Janus silver-mesoporous silica nanoparticles (FA-JNPs@ICG) by a modified Stöber method (Wang et al., 2017c) (Figure 6A). FA-JNPs@ICG had uniform ball-stick structures consisting of silver spheres and silica rods (Figure 6B). The negatively charged ICGs were loaded on the surface and in the nanochannels since the positively charged amino groups were present in the silica body. After internalization by the cancer cells, these nanoparticles employed ICGs as the effector for photothermal therapy to activate the chemotherapeutic agent. The subsequently released silver ion response to NIR irradiation could induce apoptosis in liver cancer cells as efficiently as chemotherapy. Collectively, these nanoparticles exhibited synergistic therapeutic capabilities by integrating chemotherapy and photothermal therapy. In the *in vitro* study, FA-JNPs@ICG were found to be toxic toward liver cancer cells rather than normal liver cells, with an inhibition rate of ∼90% (Figure 6C). Moreover, when exposed to NIR irradiation, the designed nanosystems exhibited a promising tumor growth inhibition index of 88.9% after 16 days of treatment in a mouse model (Figure 6D). Therefore, these nanoparticles could be a promising approach for chemo/photothermal therapy with high efficiency and safety for HCC. SNF modifications have considerably improved the effects of traditional and targeted chemotherapeutic agents, and novel SNFs that exert special antitumor effects without loading antitumor drugs are worth exploring.

### SNF-Based Ferroptotic Therapy

Ferroptosis, as a new form of programmed cell death, is iron-dependent and different from apoptosis, necrosis, and autophagy. The main process of ferroptosis is the accumulation of ROS and the subsequent redox dysfunction (Yagoda et al., 2007). In the presence of Fe^2+/3+^ ions, membrane polyunsaturated fatty acids (PL-PUFA-OH) are oxidated by lipoxygenases and/or ROS to produce lipid peroxides (PL-PUFA-OOH) (Cheng and Li, 2007), which can be converted into toxic lipid-free radicals (Seiler et al., 2008). Then, membrane rupture and cell death are caused by the lipid-free radicals (Cheng and Li, 2007). Since the ferroptotic process is rapid and intense without multi-level signal transduction unlike apoptosis and immune responses, it has garnered considerable attention (Fei et al., 2020b). When ferroptosis-inducing strategies and ferroptosis-based nanotherapeutics were integrated for HCC therapy, remarkable tumor suppression was achieved during the last decade (Ou et al., 2021). In all the attempts to explore ferroptotic treatment, metal elements such as iron, Mn, and copper, etc., have been found to be superior in inducing tumor cell ferroptosis, promoting ROS production, and interfering with cell communication when acting as Fenton or Fenton-like biocatalysts (Huo et al., 2019; Lin et al., 2019).

**FIGURE 7:**
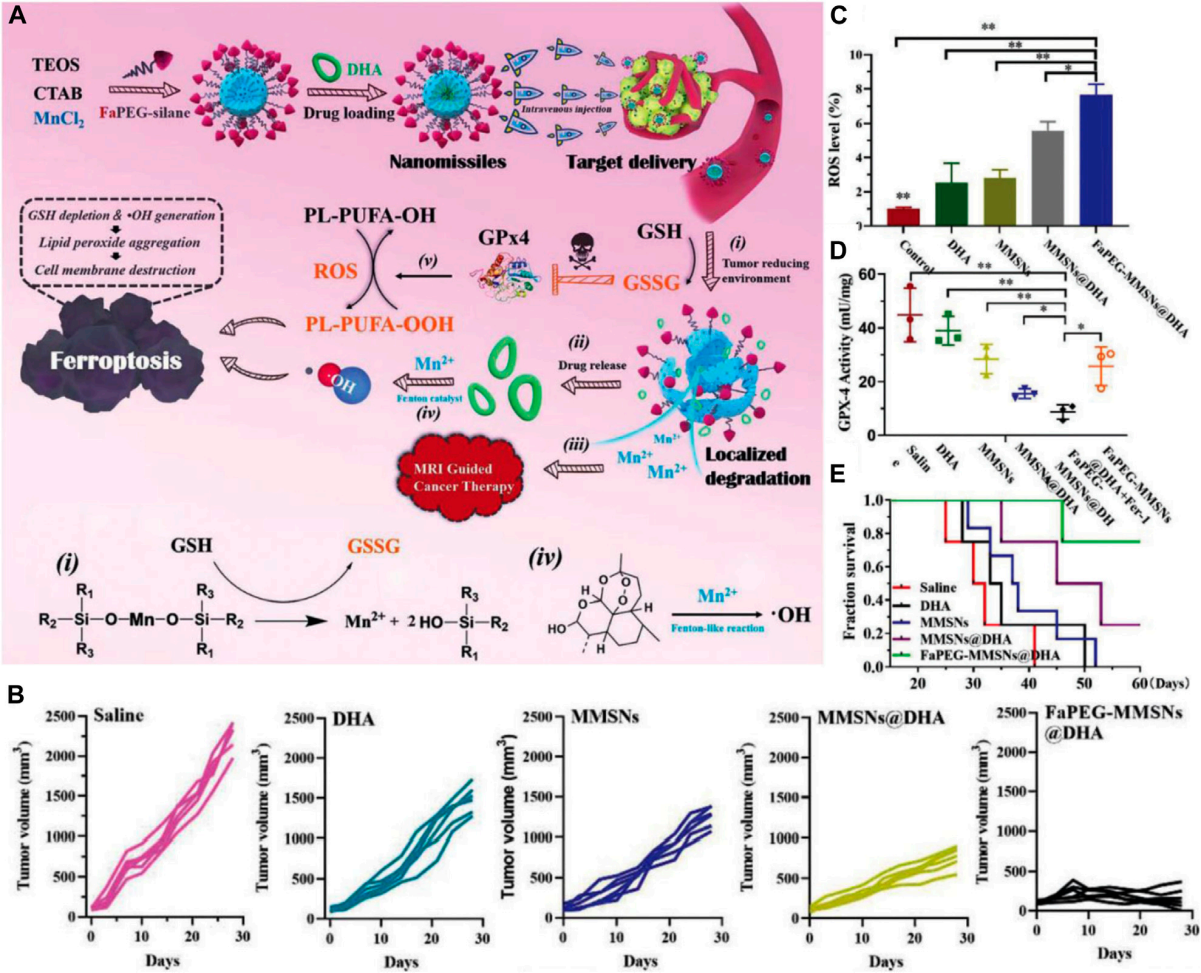
**(A)** Schematic illustration of the construction and ferroptosis-inducing mechanism of FaPEG-MMSNs@DHA (described as nanomissiles). **(B)** Time-dependent HepG_2_ tumor growth profiles. **(C)** Enhanced ROS generation in the tumor site. **(D)** GPx4 activity in tumors after different treatments. **(E)** The survival curve of mice after various treatments. Reprinted with permission from Fei et al. (2020a). Copyright (2020) Royal Society of Chemistry.

To explore new strategies for HCC therapy, our group developed Mn-doped MSNs (MnMSNs) in 2019 (Tang et al., 2019). We discovered that the manganese-oxygen chemical bonds (-Mn-O-) acted as an oxidation/reduction bond and were cleaved in malignant areas containing GSH (2–20 mM). In the acidic TME, Mn^2+^ was released after -Mn-O- cleavage, whereas intracellular GSH was consumed simultaneously. GSH consumption induced by MnMSN degradation inhibited the activity of glutathione peroxidase 4 (GPx4), thereby decreasing the accumulation of lipid-free radicals to prevent the occurrence and development of ferroptosis. Subsequently, dihydroartemisinin (DHA) was loaded onto MnMSNs (described as nanomissiles) to explore the efficiency of ferroptotic therapy that combined ROS generation and GSH depletion strategies (Figure 7A) (Fei et al., 2020a). Our *in vitro* results showed that the nanomissiles were degraded by GSH, leading to the release of DHA and the cleavage of -Mn-O-. The released Mn^2+^ interacted with GSH to induce a Fenton reaction. As a result, such degradation in the system led to GSH exhaustion, whereas ROS generation was driven by DHA. As shown in Figure 7B, the mean tumor volume in the nanomissile group was significantly decreased (168.83 ± 110.50 mm^3^) and the tumor inhibition rate was up to ∼92.56 wt%, showing better antitumor effects than those in the saline, free DHA, MnMSN, and MnMSNs@DHA groups. The ROS content was determined in the fresh tumor tissue obtained from each group to explore the ferroptotic effect of nanomissiles *in vivo*. As showed in Figure 7C, the nanomissile group exhibited the highest ROS generation in HCC tumors. Moreover, the nanomissiles inhibited the activity of GPx4 the most compared with that in other groups (Figure 7D). As an N-cyclohexyl compound, Fer-1 acted as a lipophilic anchor within biological membranes to prevent the accumulation of PL-PUFA-OOH, thereby inhibiting the occurrence of ferroptosis induced by FaPEG-MnMSNs@DHA (Figure 7D). These results suggested the excellent ferroptosis-inducing effect of nanomissiles *in vivo*. Moreover, the mice in the nanomissile group were still alive after 41 days of treatment, whereas those in the control group were dead (Figure 7E). In general, our studies demonstrated an SNF-involved, ferroptosis-based therapeutic strategy that is expected to be a candidate for the next generation of HCC therapy. Apart from the Mn ion discussed here, iron or copper ions that can act as Fenton or Fenton-like biocatalysts can be used as tumor ferroptosis inducing agents (Peng et al., 2013; Shi et al., 2016).

### SNF-Based Radiotherapy

**FIGURE 8:**
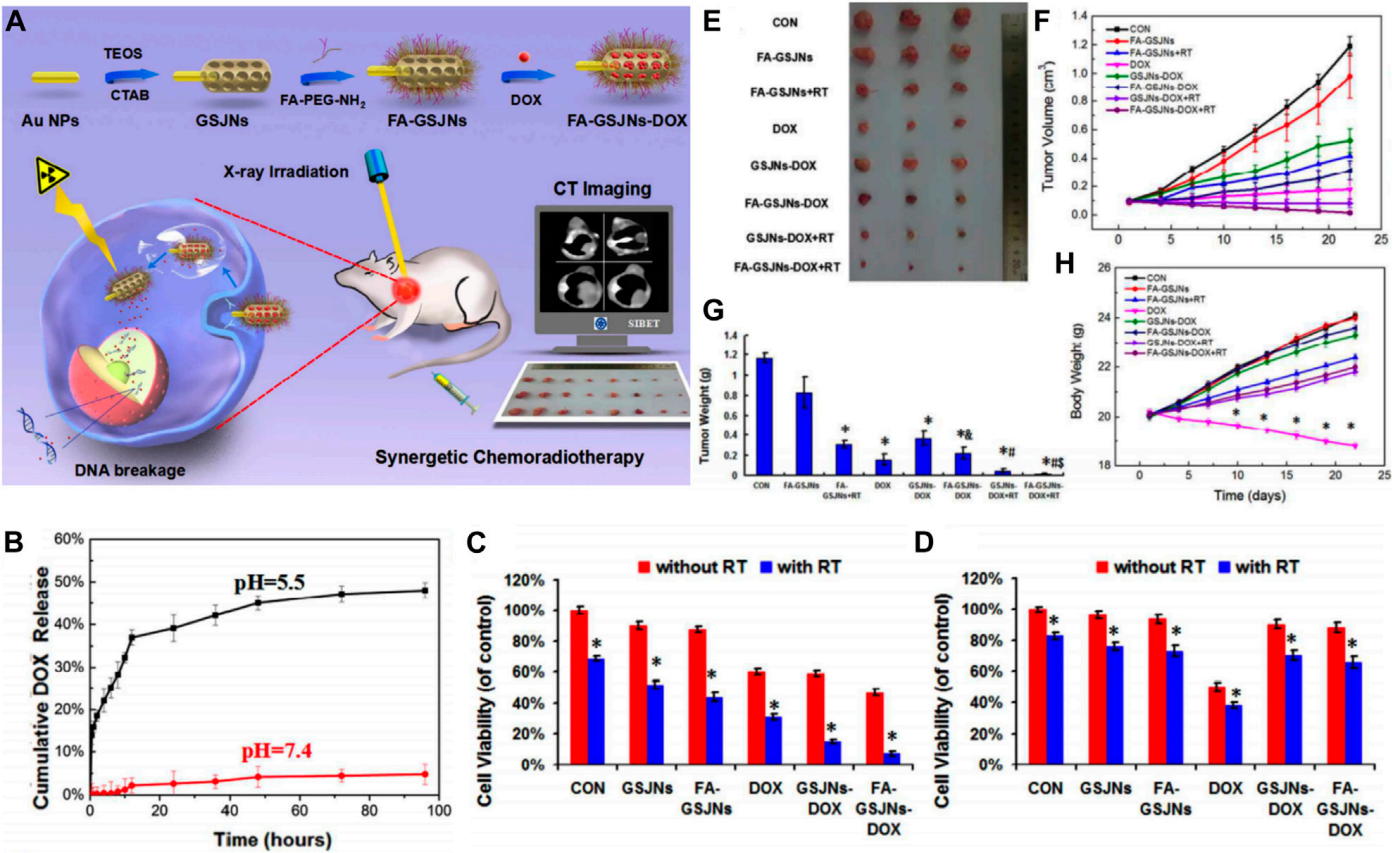
**(A)** Schematic illustrations of the synthetic procedure for the DOX-loaded Au-mesoporous silica Janus NPs and application for synergetic chemoradiotherapy and CT imaging in HCC theranostics. **(B)** pH-dependent drug-release profiles of FA-GSJNs-DOX. Cytotoxic profile of FA-GSJNs-DOX against **(C)** SMMC-7721 cells and **(D)** HL-7702 cells for 24 h. *In vivo* chemoradiotherapy effect: **(E)** Tumor photographs, **(F)** volume, **(G)** weight, **(H)** body weight from mice in each group over 23 days. Reprinted with permission from Wang et al. (2017d). Copyright (2017) American Chemical Society.

Radiotherapy (RT) is the standard clinical treatment based on radiation. While high-energy radiation eradicates the tumor cells, it also inevitably causes damage to normal tissues, resulting in serious side effects such as gastric dysfunction and bone marrow suppression. With the rapid development of nanotechnology, new radiosensitizers combining radiotherapy and nanotechnology may have high efficiency and safety for HCC treatment. Among them, Au-mesoporous silica Janus nanoparticles (GSJNs) were engineered by a modified sol-gel method (Figure 8A) (Wang et al., 2017d). Tetraethoxysilane (TEOS) was used as a silica source, cetrimonium bromide (CTAB) was used as a template, and the AuNPs were used as a substrate (Wang et al., 2015; Wang et al., 2016). The Janus NPs were formed by combining Au NPs with silica NPs together through one single unit. The modification of the framework inside MSNs was different from that in metal-dopped MSNs. While metal-dopped MSNs constitute a metal element doped into the structure of -Si-O-Si-, Janus NPs constitute metal NPs hybridized with silica NPs to form a single carrier. They are two different ways of framework modification but they are both useful to enable the synthesized SNFs with novel capacities such as high drug-loading, biodegradability, and tumor targeting. In this system, a Janus nanostructure representing an asymmetrical shape was designed to increase radiation efficiency and have sufficient surface area available for anticancer agent loading. Au can be used as a radiosensitizer to improve the radiotherapeutic effect for their high radiation absorption. Enhanced HCC targeting was achieved by conjugating folic acid (FA) onto the surface of GSJNs. After conjugation, the FA-GSJNs were endocytosed accessibly by cancer cells more than normal cells and smartly released drugs in acidic endo-lysosomes. Through the protonation and dissociation of amine groups, DOX was released from the GSJNs in a pH-responsive pattern (Figure 8B). With the assistance of X-ray irradiation, the FA-GSJNs-DOX exhibited more pronounced cell viability inhibition than the FA-GSJNs-DOX or RT alone toward HCC cells after 24 h of incubation (Figures 8C,D). Furthermore, the combination between FA-GSJNs-DOX and X-ray irradiation resulted in the highest tumor growth inhibition than in other groups after 23 days of treatment in nude mice bearing SMMC-7721 xenografts (Figures 8E–H). Notably, no obvious weight loss or cardiotoxicity was observed in mice from this group as seen with DOX treatment. In general, this novel nanosystem integrated with dual functionalities turned out to be a promising treatment for HCC therapy. In another study, tirapazamine (TPZ)-loaded Janus gold triangle-MSNs (FA-GT-MSNs@TPZ) were synthesized to improve the unsatisfactory therapeutic effects caused by the hypoxic microenvironment inside HCC (Wang et al., 2019). There was a silver triangular gold head connected to an extended silicon connector. The gold nanotriangels integrated RT and photothermal (PTT) as the dual-therapeutic agent. TPZ, as a hypoxia-activated prodrug, generated oxidizing radicals in low pH and low oxygen pressure conditions, which were usually observed in the TME. After NIR and X-ray exposure, FA-GT-MSNs@TPZ treatment killed almost all SMMC-7721 cells in hypoxia. Notably, in the *in vivo* study, tumor growth was completely inhibited when FA-GT-MSNs@TPZ with X-ray and NIR were used in tumor-bearing mice. In general, metal-silica Janus nanoplatforms were capable of transforming external energy to antitumor effects. Although recent studies in radiated and NIR energy are limited, more multifunctional metal-silica Janus nanoplatforms using different kinds of energies such as magnetic, electric, and sound energy are expected to be developed for HCC therapy in the future. While recent studies focused on the search for effective radiosensitizers, metal-silica hybridized nanoparticles may realize the possibility of becoming self-radiated emission agents, which exert radiotherapeutic effects without X-ray radiation, thus minimizing the side effects associated with radiation.

### SNF-Based Phototherapy

#### SNF-Based Photodynamic Therapy

Photodynamic therapy (PDT) has become a promising clinical therapy for patients with advanced cancers (Agostinis et al., 2011). It combines light at appropriate wavelengths with light-triggered photosensitizers to cause selective damage to the target tissue (Kwiatkowski et al., 2018). When applied to cancer treatment, PDT excels as a minimally invasive therapy with various benefits. To date, PDT has been successfully used to treat HCC, cholangiocarcinoma, hepatoblastoma, and liver metastases (Zou et al., 2020). The clinical outcomes (Bahng et al., 2013; Muragaki et al., 2013; Yanovsky et al., 2019) suggested that it would become an encouraging therapy to prolong overall survival and enhance the quality of life for patients with HCC. The biological mechanism of PDT in killing tumor cells depends on the following ways (Hou et al., 2020): 1) the direct necrosis effect on tumor cells, 2) apoptosis caused by singlet oxygen (produced by photodynamic), and 3) immunogenic cell death stimulated by photodynamic in dying or dead cells. However, organic molecules are typically used as photosensitizers, such as porphyrins, curcuminoids, phenothiazines, and xanthene, which have disadvantages such as off-targeting, instability *in vivo*, and π–π superposition-induced quenching (Cai et al., 2018). These defects may attenuate the therapeutic effects and generate side effects in patients. Recent studies have shown that the integration of photosensitizers with nanoparticles can enhance therapeutic efficiency and also reduce their side effects (Guo et al., 2018; Han et al., 2018). Many nanoparticulate vectors have been suggested for PDT, particularly silica-based nanostructures (Chatterjee et al., 2008; Cheng and Lo, 2011; Li et al., 2021b). MSNs functionalized with photosensitizers have received considerable attention for use in PDT since 2009 (Couleaud et al., 2010). Integrating the photosensitizers inside the pores or skeleton of the MSNs can improve the selectivity of PDT to tumor cells and enhance its therapeutic effect. Core-shell and finer nanostructure-based MSNs have been synthesized for highly efficient PDT for HCC treatment (Liu et al., 2014; Wong et al., 2017).

**FIGURE 9:**
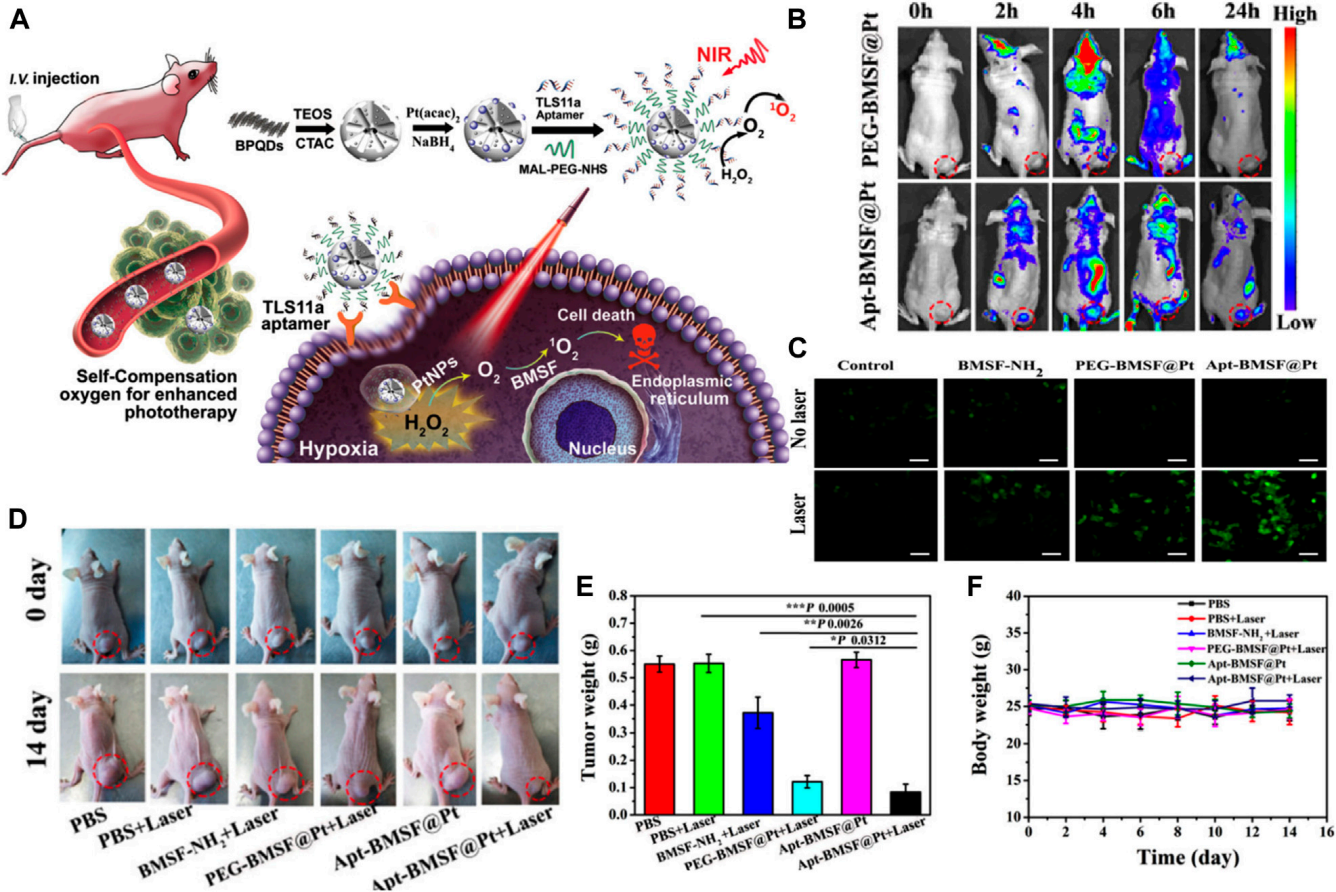
**(A)** Schematic illustration of Apt-BMSF@Pt and the photocatalysis mechanism for enhancing PDT. **(B)** *In vivo* fluorescence imaging of PEG-BMSF@Pt and Apt-BMSF@Pt at different time points after intravenous injection. **(C)** The DCFH-DA detection of intracellular ROS in HepG_2_ cells after indicated treatment. **(D)** *In vivo* response to PBS, BMSF-NH2, PEG-BMSF@Pt, and Apt-BMSF@Pt with or without laser irradiation. **(E)** The average tumor weight of mice after indicated treatments (*n* = 5); **(F)** Mean body weight of mice during indicated treatments. Reprinted with permission from Lan et al. (2019). Copyright (2019) American Chemical Society.

Lan et al. constructed a black phosphorus quantum dot (BPQDs)-hybridized mesoporous silica framework (BMSF) that consisted of the aptamer “TLS11a”-decorated nanocatalyst for HCC-specific targeting and self-compensate oxygen (O_2_) for the hypoxic tumor microenvironment to improve the efficiency of PDT (Figure 9A) (Lan et al., 2019). The remarkable photocatalysis in PDT and the broad photoabsorption property from UV to NIR of BPQDs has attracted attention from researchers (Gui et al., 2018). However, the instability of BPQDs in a water-oxygen coexistence environment hindered their clinical use, since the consequent degradation would reduce the PDT efficacy. Therefore, the BMSF was developed for more efficient PDT. These results suggested that the designed BMSF could not only take advantage of the photocatalysis capacity of BPQDs but also gain the extra ability to protect BPQDs from oxidation and degradation. The PDT effect can be compromised when hypoxia and hydrogen peroxide accumulated in the tumor microenvironment, especially in solid tumors like HCC (Liu et al., 2018a). Considerable efforts have been made to alleviate hypoxia by nanotechnology. Generating oxygen *in situ* may be one of the feasible methods to relieve hypoxia (Wang et al., 2017a; Yu et al., 2018). In this case, the decomposition of H_2_O_2_ can produce oxygen in the presence of catalase or catalase-like materials. The BMSF was prepared with *in situ*-synthesized Pt nanoparticles (Apt-BMSF@Pt) by a one-step method that converted H_2_O_2_ into O_2_ for increasing the singlet state of O_2_ generation (Figure 9C) (Lan et al., 2019). As shown in Figure 9B, by combining with the TLS11a aptamer, which was a powerful molecular targeting element with promising thermal stability and immunogenicity, the Apt-BMSF@Pts were capable of HCC targeting *in vivo* specifically, and the efficient tumor growth inhibition was achieved by enhanced PDT (Figure 9D). More strikingly, the tumor growth at day 12 was completely suppressed when the tumor-bearing mice were treated with Apt-BMSF@Pt and laser irradiation (Figures 9E,F). In another study, Lv et al. developed GdOF: Ln@SiO_2_-ZnPc-CDs-FA UCL microcapsules (UCMCs) (Lv et al., 2015). It was core-shell, yolk-like microcapsules in which Ln was used as cores and mesoporous silica layer as shells, and there were large mesoporous pores between the core and shell. These silica shells enabled the microcapsules with a large surface area for DOX loading, good biocompatibility, and easy modification with functional groups. When exposed to NIR irradiation, the tumors in H22 mice treated with UCMCs were inhibited remarkably and even stopped growing, suggesting a synergy in the chemo-photodynamic treatment. SNF-mediated targeted drug-delivery system is a novel PDT approach that increases the tumor specificity of photosensitizers and decreases their cytotoxicity. On the other hand, the combination of PDT and chemotherapy through the new SNF system could offer a promising direction for the use of PDT in HCC treatment.

#### SNF-Based Photothermal Therapy

**FIGURE 10:**
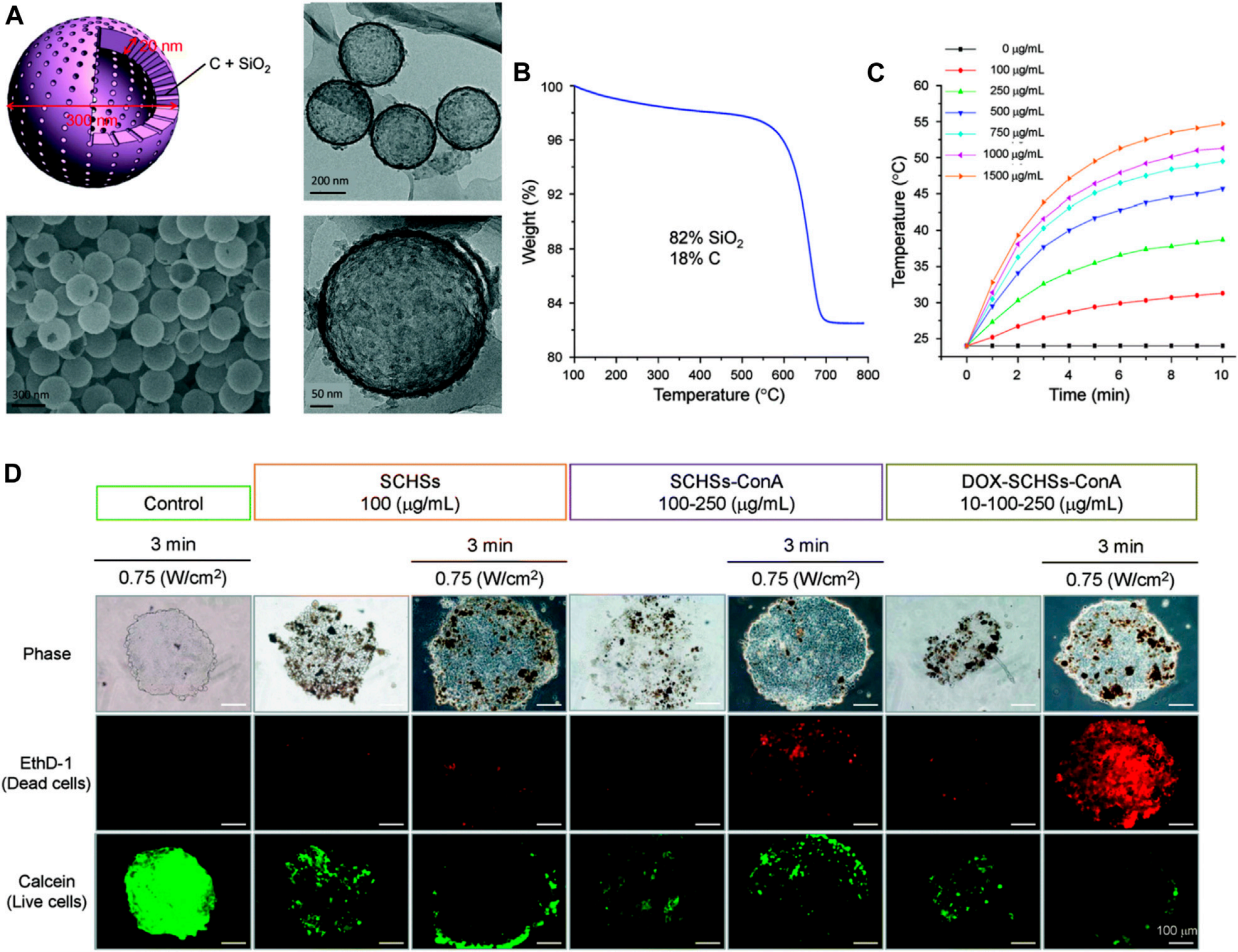
**(A)** Morphology of a silica–carbon hollow sphere. **(B)** Thermogravimetric analysis (TGA) curve showing the thermal decomposition of SCHSs in air. Approximately 82% mass of silica (SiO_2_) remains at a temperature of 800°C. **(C)** Temperature-time curves for media under NIR illumination for various concentrations (100–1,500 μg ml^−1^) of SCHSs. **(D)** Cell viability of chemo-photothermal treatment on *in vitro* 3D MCTSs, representative fluorescence images of calcein AM and EthD-1 co-stained Huh-7 cells. Reprinted with permission from Chen et al. (2018). Copyright (2018) Royal Society of Chemistry.

Recently, the potential application of MSN-based photothermal therapy (PTT) and their combinational therapy in treating HCC have been explored (Chen et al., 2015c; Li et al., 2021a; Yang et al., 2021). PTT uses the deep tissue penetration and good biocompatibility of near-infrared light (NIR 700–2500 nm) to irradiate the pathological tissue, and the photothermal material converts the light energy into heat, leading to a localized increase in temperature. The photothermal damage of tumor cells usually begins when the local temperature reaches 41°C (Xie et al., 2011). Relying on the precise irradiation of near-infrared light and the efficient photothermal conversion ability of nanomaterials, PTT is expected to become a minimally invasive or even noninvasive cancer treatment. Chen et al. synthesized silica-carbon hollow spheres (SCHSs) *via* a surface activation method (Chen et al., 2015c). Carbon black absorbs NIR to generate heat in the area of concern and the silica skeleton constructs hollow spherical nanostructures that can effectively prevent heat loss; so, nanoparticles can be maintained at high temperature for a relatively long time, thereby inducing enhanced PTT efficacy. In this study, nanoscale ConA-SCHSs (nearly 300 nm) with a shell thickness of about 15–20 nm selectively induced considerable necrosis and apoptosis in HCC cells (ML-1 and Huh-7) after 808 nm NIR irradiation. The biocompatible and highly concentrated water-based dispersions of SCHSs were ideal photothermal nanotherapeutics. Furthermore, Chen et al. continued to embed the antitumor compound DOX into SCHSs by combining heat and vacuum to improve antitumor efficacy (Chen et al., 2018). As shown in Figure 10A, the synthesized DOX-loaded SCHSs were in a uniform and narrow size distribution (≈300 nm) with a high surface area (150 m^2^g^−1^), and DOX-encapsulation efficiency (87%). These nanoparticles exhibited both superior heat-accumulation ability after 808 nm NIR illumination and enhanced DOX antitumor activity (Figures 10B,C). DOX-SCHSs-ConA was further applied in an *in vitro* 3D tumor model (MCTS), which was an intermediate assay between cell culture and animal studies. The results (Figure 10D) indicated that NIR irradiation caused considerable damage to MCTS with SCHSs, and DOX-SCHSs-ConA inhibited tumor cells the most under all the studied conditions. The SNFs integrated PTT and chemotherapy in a single platform, and could make the best use of the synergistic effects and reduce the side effects by accurately targeting the chemotherapeutic agents and heat to the tumor tissues. While novel photosensitizers are being developed, SNFs integrating multiple modalities could improve the therapeutic effect of PTT and be considered for practical application in a clinic setting.

### SNF-Based Sonodynamic Therapy

**FIGURE 11:**
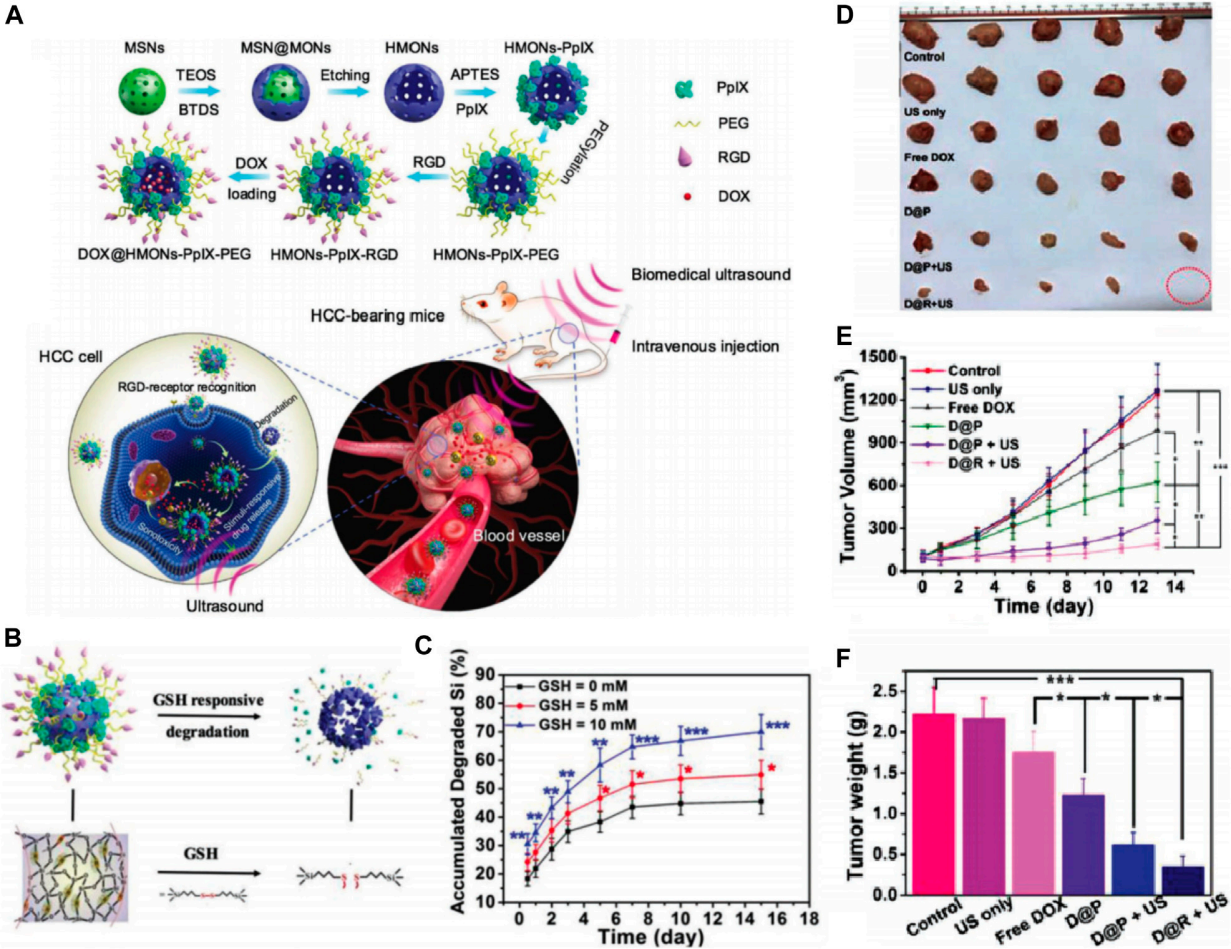
**(A)** Schematic illustration of the synthetic process of HMONs-PpIX-RGD and synergistic chemo-SDT against HCC tumor xenograft on nude mice. **(B)** Schematic illustration of microstructure and corresponding biodegradation induced by breaking up of physiologically active disulfide bond within the framework of HMONs-PpIX-RGD. **(C)** The corresponding amount of degraded Si component during 15 days observation. *In vivo* synergistic chemotherapy-SDT against HCC tumor-bearing mice: **(D)** *Ex vivo* tumor images of different treated groups as indicated. **(E)** time-dependent tumor growth curves after different treatments and **(F)** tumor weights of mice on the 15th day after the treatments. Reprinted with permission from Li et al. (2018). Copyright (2018) John Wiley and Sons.

The underlying principle of sonodynamic therapy (SDT) is the combination of US irradiation and sonosensitizers to generate ROS like ^1^O_2_, which leads to cancer cell death depending on the redox status. SDT not only inherits a high tissue penetration depth of US but also enhances the sensitivity of tumor cells to chemotherapy. Various sonosensitizers including organic and inorganic molecules have been adopted for SDT, but some issues such as low chemical/biological stability, poor tumor targeting, and low biodegradation limit their transition to clinical application (Son et al., 2020). Thus, novel sonosensitizers with high biodegradability and chemical/biological stability need urgent exploration. With specific modifications inside the framework of MSNs, novel silica-based nanoplatforms were developed with enhanced biodegradability, biocompatibility, and higher drug-loading capacity. Li et al. developed hollow mesoporous organosilica nanoparticles (HMONs) with an organic-inorganic hybrid framework (Li et al., 2018). These HMONs were developed based on the chemical homology strategy using MSNs as the hard template and mesoporous organosilica layer were coated on the surface (Figure 11A). The organic groups referred to as disulfide bonds in the framework facilitated easy biodegradation behavior in the TME, thereby improving biocompatibility and biosafety of the nanoparticles. The inorganic mesoporous silica groups in the framework offered a larger surface area, and sufficient surface chemistry enabled the covalent anchoring of protoporphyrin (PpIX, HMONs-PpIX) into the mesopore surface and noncovalent hydrophobic-hydrophobic interaction with DOX. Furthermore, the surface conjugated RGD enabled active tumor targeting in the case of HCC and facilitated a stimuli-responsive release (Figures 11B,C). The DOX@HMONs-PpIX-RGDs were used as effective nanosonosensitizers and produced with efficient ^1^O_2_ when triggered by the US, leading to the apoptosis of HCC cells. As expected, when exposed to ultrasound irradiation, these nanoparticles exhibited desirable sonotoxicity and chemotoxicity toward SMMC-7721 cells. Moreover, a synergistic therapeutic effect was observed when DOX@HMONs-PpIX-RGD plus ultrasound irradiation were used to treat nude mice bearing SMMC-7721 tumor cells (Figures 11D–F). The tumor inhibition rate was 84.7%. Thus, these nanoparticles are expected to be a promising therapy for HCC. Inorganic sonosensitizers, especially the SNFs, holds immense potential for enhancing the stability, biodegradation, and therapeutic effects of HCC treatment.

### SNF-Based Immunotherapy

**FIGURE 12:**
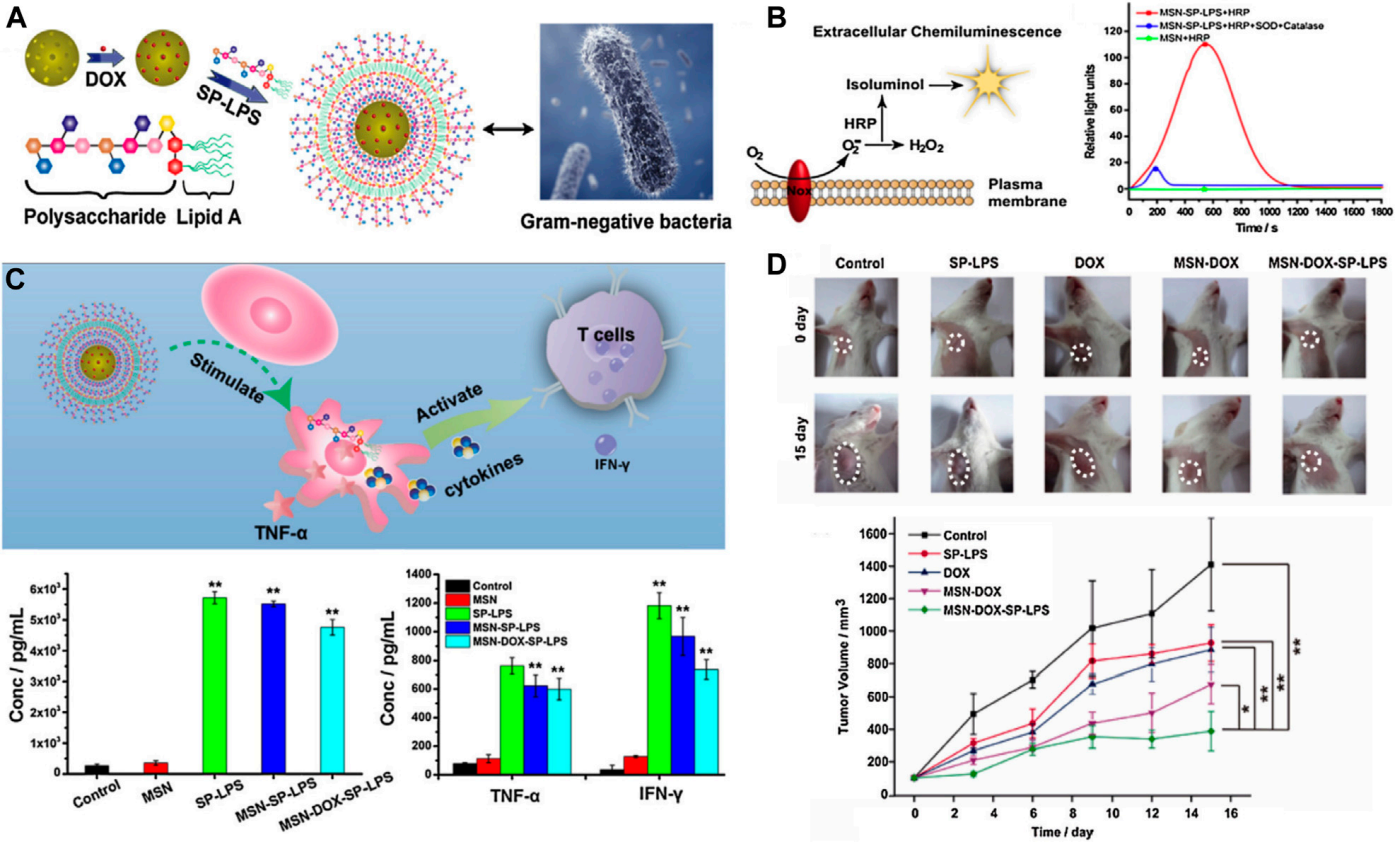
**(A)** Schematic illustration of Gram-negative mimicking MSP-DOX-SP-LPS; **(B)** MSP-SP-LPS stimulate splenocytes to produce extracellular ROS; **(C)** Schematic illustration of MSP-DOX-SP-LPS activating macrophages and subsequently stimulating T cells. The picture of tumors after different treatments at 0 and 15th day and the location of the tumors was labeled by broken circles. **(D)** And the relative tumor volumes were treated with different samples. Reprinted with permission from Dong et al. (2017). Copyright (2017) Elsevier.

Immunotherapy has also made some progress in HCC treatment. For example, in 2017, nivolumab and pembrolizumab were approved by the FDA as the second-line treatment for advanced HCC. In May 2020, the programmed cell death 1 ligand (PD-L1) antibody atelizumab combined with the target drug bevacizumab was introduced as the first-line treatment for advanced HCC, which reduced the risk of death in these patients by 42% compared with the traditional first-line treatment using sorafenib, thus bringing new hope to the long-term survival of patients with HCC (Casak et al., 2021). As one of the most widely investigated nanocarriers, the immunological effect of mesoporous silica nanoparticles in HCC treatment has been concerned and investigated. A pathogen-mimicking system based on detoxified lipopolysaccharide-modified mesoporous silica nanoparticles was designed to deliver DOX for synergistic chemo-immunotherapy of HCC (Figure 12A) (Dong et al., 2017). The pathogen-mimicking nanosystem (MSP-DOX-SP-LPS) recruited phagocytes such as neutrophils and macrophages, when injected into the tumor sites, thus simulating the pathogen infection. The recruited neutrophils generated a vast amount of extracellular ROS (Figure 12B) to trigger the loaded DOX release due to the boronic esters between MSN and the lipopolysaccharide (LPS), which could respond to the ROS, and thus, achieved the chemotherapy of HCC. The recruited macrophages could be activated by the MSN-SP-LPS to attack tumor cells directly by secreting tumor necrosis factor-α (TNF-α) or subsequently activating T cells for initiating the antitumor immune response (Figure 12C). This chemo-immunotherapy combination strategy showed an obvious synergistic therapeutic effect with inconspicuous side effects in the HCC mice model. The mice treated with MSP-DOX-SP-LPS produced a significant immune memory, showing lower recurrence of the primary tumor and lower growth rate of reinoculated tumor (Figure 12D) (Dong et al., 2017). In summary, SNF-mediated immunotherapy is effective for HCC treatment. The rapid advancement of immunotherapy based on nanoparticles holds potential for recent new approaches such as antitumor vaccines, immune checkpoint blockade, and immunogenic cell death (Liu et al., 2019b). On the other hand, the concept of realizing chemo-free treatment for cancer therapy is emerging (Zhu, 2020).

## Conclusion and Perspectives

Recently, several nanopharmaceuticals are undergoing further clinical trials for HCC treatment, and numerous products are being developed that are expected to benefit patients at different stages of HCC (van der Meel et al., 2017). Nanotechnology, by overcoming existing problems, has made considerable progress in the diagnosis and treatment of HCC. Nanomaterials such as liposomes, organic microspheres, and silica-based nanocarriers have been extensively investigated and have immense potential for clinical use, owing to their good biocompatibility and biodegradability. Since 2011, research on the concept of silica-based nanomaterials for the diagnosis and treatment of HCC has increased. Compared with other drug carriers, large specific surface areas and pore volumes make silica-based nanomaterials stellar nanovehicles in the field of drug delivery. Inorganic small-molecule drugs, organic drugs, and gene fragments can all be loaded by the mesoporous structure of silica-based nanomaterials. The sustained and controlled release features of MSNs could stabilize the drug concentration in the body and extend the dosing intervals. In addition, easy surface modification of MSNs can promote the development of active targeting and tumor-specific, drug-release nanoagents against HCC. Scientists have summarized the advantages of MSNs as drug carriers against HCC (including therapeutic and diagnostic agents) (Tao et al., 2020). However, the attractiveness of silica-based nanomaterials is not limited to the field of drug delivery (Kumar et al., 2018; Živojević et al., 2021). Skeleton structure modification of the ordinary MSNs will confer novel characteristics for the diagnosis and treatment of HCC.

During the past decades, rapid progress in materials science has further promoted the development of silica-based nanomaterials. Scientists in the field of nanomaterials add inorganic metal ions or small organic molecules in the development process of MSNs starting from crystal nuclei into nanosized particles, which can increase the porosity of MSNs, accelerate their degradation in the body, and reduce accumulation toxicity. These metal- or small molecule-doped silica-based nanomaterials can exhibit special antitumor effects without loading antitumor drugs. Surface chemists have synthesized silica-based Janus nanomaterials through the directional growth of silica sources on the surface of other materials. These nanomaterials often exhibit special physical and chemical properties against HCC, such as strong photothermal- and radiation-conversion abilities. Importantly, these novel silica-based nanomaterials still have highly ordered mesoporous structures that can efficiently load and sustain the release of drugs in HCC treatment. Therefore, the novel silica-based nanomaterials summarized in this review often have multiple therapeutic effects against HCC. To reflect the diversified functions of silica-based nanomaterials in addition to drug delivery, in this review, we outlined silica-based nanoframeworks involved in the diagnosis and treatment of HCC. Compared with ordinary MSNs loaded with diagnostic and therapeutic agents, new silica-based nanodrugs have obvious advantages in the HCC theranostics. Despite the enthusiasm for the efficiency and potential clinical applications of new silica-based nanoframeworks, the following points need to be noted:

First, the doping theory of silica-based nanomaterials has not yet been finalized. Small molecules that can be doped into the framework of MSNs are still in the exploratory stage. Therapeutic drugs or diagnostic agents with appropriate molecular structures and sizes can be doped into the framework structure of mesoporous silica, thereby enriching the functions of silica-based nanomaterials. However, the release of small molecules from the framework should be investigated because most drugs only exert their effects after dissolution.

Second, the function of metal-doped or metal-hybrid MSNs in HCC therapy needs further development, and studies should focus on the synergistic therapeutic effect of the combined use of novel silica-nanoplatforms and the loaded antitumor drugs. Many metal-doped MSNs have been designed and synthesized but few studies have explored their role in the diagnosis and treatment of HCC. For instance, calcium-doped MSNs can be rapidly degraded in an acidic microenvironment (Hao et al., 2015). The fragmentation of calcium-doped MSNs releases the loaded drugs and calcium ions. The released calcium is expected to cause calcification in HCC tissues. Thus, calcium-doped MSN-based nanodrugs could play multiple roles in cancer therapy. However, there is a lack of research to verify its role in the treatment of HCC. Iron-doped MSNs can release iron ions in the acidic tumor microenvironment (Peng et al., 2013; Shi et al., 2016), and iron ions can act as Fenton catalysts to induce ferroptosis in tumor cells (Huo et al., 2019). Furthermore, the free iron ions can act as MRI agents for tumor diagnosis (Liu et al., 2018b). However, the diagnostic and therapeutic effects of the iron-doped MSNs on HCC also lack verification. Other novel silica-based nanomaterials such as metal-silica hybrid nanomaterials are formed by the directional growth of silica sources on the metal surface. A few studies have reported metal-silica hybrid nanomaterials, mainly including gold- and silver-silica Janus nanomaterials. With extensive studies in the future, more cost-effective and efficient energy conversion materials, such as metal sulfides and metal oxides, can be combined with MSNs so that they can play a more powerful role in the treatment of HCC.

Third, the biocompatibility of the novel silica-based framework should be explored further, and the degradation feature and toxicity of its metabolites need to be investigated. Researchers should investigate the effects of metal elements or molecules on the biocompatibility of novel silica-based nanomaterials to avoid undesirable harm to normal tissues. The -Si-O-Si-skeleton structure in traditional MSNs is stable and degrades very slowly in the body (more than 3 weeks) (Alexis et al., 2008; Shi et al., 2016), and is likely to accumulate in important organs like the liver, kidneys, lungs, and bladder (He and Shi, 2011; Huang et al., 2011), thereby causing severe inflammation, oxidative damage, organ fibrosis, and other adverse reactions (Nabeshi et al., 2011a; Nabeshi et al., 2011b). Therefore, the degradation and metabolic properties of novel silica-based nanomaterials in the body should be explored. Thus, silica-based nanodrugs can help achieve high precision serum molecular biology and HCC imaging results, and assist in individualized management. We believe that more upcoming novel silica-based nanomaterials can build new strategies for HCC therapy.

## Glossary

HCC: hepatocellular carcinoma
MDR: multidrug resistance
MSNs: mesoporous silica nanomaterials
RGD: arginine-glycine-aspartic acid
TME: tumor microenvironment
nDDS: nano drug delivery systems
SNFs: silica-based nanoplatforms
Mn: manganese
MnMSNs: Mn-doped mesoporous silica nanocarrier: Mn-doped MSNs
MRI: magnetic resonance imaging
GSH: glutathione
PET: positron emission tomography
AFP: alpha-fetoprotein
SERR: surface-enhanced resonance Raman
SERRS: SERR scatting
EDTA·2Na: ethylenediaminetetraacetic acid disodium salt
AuNPs: gold nanoparticles
SiO_2_ NPs: silica nanoparticles
RIfS: reflectometric interference spectroscopy
SiN: silicon nitride
Mn-SiO_2_: Mn^2+^-doped SiO_2_ nanoparticles
AMSNs: arginine-rich manganese silicate nanobubbles
TEM: ransmission electron microscopy
Arg: arginine
RES: reticuloendothelial system
PAI: photoacoustic imaging
NIR: near infra-red
PVMSNs: polydopamine-doped virus-like mesoporous nanoparticles
GO: graphene oxide
rGO@PVMSNs: PVMSNs coated reduced graphene oxide
PA: photoacoustic
HA-SiNP: hyaluronate-silica nanoparticle
FITC: fluorescein isothiocyanate
FSNPs: FITC-doped silica nanoparticles
SA: streptavidin
SA-FSNPs: FSNPs conjugated with SA
Bio-TLS11a: biotin-labeled aptamer TLS11a
US: ultrasound
GPC-3: glypican-3 protein
F-MSN: perfluorodecyltriethoxysilane-modified MSNs
M-MSNs: trimethylchlorosilane-modified MSNs
P-SS: perfluorodecyltriethoxysilane-modified silica nanoparticles
INBs: interfacial nanobubbles
DOX: doxorubicin
Cyc c: cytochrome c
YMSNs: mesoporous silica nanoparticles
YMSN-NBC-Cyt-c-NBC-LA@DOX: H_2_O_2_-responsive Cyt c/DOX co-delivery system
YMSN-NBC-Cyc-c-NBC-LA: H_2_O_2_-responsive Cyt c delivery system
ROS: reactive oxygen species
ICG: indocyanine green
FA-JNPs@ICG: ICG-loaded Janus silver-mesoporous silica nanoparticles
PL-PUFA-OH: polyunsaturated fatty acids
PL-PUFA-OOH: lipid peroxides
Fe: iron
GPx4: glutathione peroxidase 4
DHA: dihydroartemisinin
RT: radiotherapy
TEOS: Tetraethoxysilane
CTAB: cetrimonium bromide
GSJNs: Au-mesoporous silica Janus nanoparticles
FA: folic acid
TPZ: tirapazamine
FA-GT-MSNs@TPZ: TPZ-loaded Janus gold triangle-MSNs
PTT: photothermal therapy
PDT: photodynamic therapy
BPQDs: black phosphorus quantum dots
BMSF: BPQDs-hybridized mesoporous silica framework
Apt-BMSF@Pt: BMSF with in situ synthesized Pt nanoparticles
UCMCs: GdOF: Ln@SiO_2_-ZnPc-CDs-FA UCL microcapsules
SCHSs: silica-carbon hollow spheres
MCTS: 3D tumor model
SDT: sonodynamic therapy
HMONs: hollow mesoporous organosilica nanoparticles
PpIX: protoporphrin
PD-L1: programmed cell death 1 ligand
LPS: lipopolysaccharide
TNF-α: tumor necrosis factor-α
